# Association of combination antiretroviral therapy with risk of neurological diseases in patients with HIV/AIDS in Taiwan: a nested case-control study

**DOI:** 10.3389/fphar.2023.1110605

**Published:** 2023-06-08

**Authors:** Chen-Hsing Chou, Jian-Shiun Chiou, Mao-Wang Ho, Ni Tien, Te-Mao Li, Mu-Lin Chiu, Fuu-Jen Tsai, Yang-Chang Wu, I-Ching Chou, Hsing-Fang Lu, Ting-Hsu Lin, Chiu-Chu Liao, Shao-Mei Huang, Wen-Miin Liang, Ying-Ju Lin

**Affiliations:** ^1^ PhD Program for Health Science and Industry, College of Health Care, China Medical University, Taichung, Taiwan; ^2^ Section of Infectious Diseases, Department of Internal Medicine, China Medical University Hospital, Taichung, Taiwan; ^3^ Department of Internal Medicine, School of Medicine, China Medical University, Taichung, Taiwan; ^4^ Department of Medical Laboratory Science and Biotechnology, China Medical University, Taichung, Taiwan; ^5^ School of Chinese Medicine, China Medical University, Taichung, Taiwan; ^6^ Genetic Center, Department of Medical Research, China Medical University Hospital, Taichung, Taiwan; ^7^ Department of Biotechnology and Bioinformatics, Asia University, Taichung, Taiwan; ^8^ Department of Pediatrics, China Medical University Children’s Hospital, Taichung, Taiwan; ^9^ Graduate Institute of Integrated Medicine, China Medical University, Taichung, Taiwan; ^10^ Department of Health Services Administration, China Medical University, Taichung, Taiwan

**Keywords:** antiretroviral therapy, neurocognitive impairment, nested case-control study, cumulative defined daily dose, CNS penetration effectiveness score

## Abstract

Heterogeneous neurocognitive impairment remains an important issue, even in the era of combination antiretroviral therapy (cART), with an incidence ranging from 15% to 65%. Although ART drugs with higher penetration scores to the central nervous system (CNS) show better HIV replication control in the CNS, the association between CNS penetration effectiveness (CPE) scores and neurocognitive impairment remains inconclusive. To explore whether ART exposure is associated with the risk of neurological diseases among patients with HIV/AIDS, this study in Taiwan involved 2,571 patients with neurological diseases and 10,284 matched, randomly selected patients without neurological diseases between 2010 and 2017. A conditional logistic regression model was used in this study. The parameters for ART exposure included ART usage, timing of exposure, cumulative defined daily dose (DDD), adherence, and cumulative CPE score. Incident cases of neurological diseases, including CNS infections, cognitive disorders, vasculopathy, and peripheral neuropathy, were obtained from the National Health Insurance Research Database in Taiwan. Odds ratios (ORs) for the risk of neurological diseases were conducted using a multivariate conditional logistic regression model. Patients with a history of past exposure (OR: 1.68, 95% confidence interval [CI]:1.22–2.32), low cumulative DDDs (< 2,500) (OR: 1.28, 95% CI: 1.15–1.42), low adherence (0 < adherence (ADH) ≤ 0.8) (OR: 1.46, 95% CI: 1.30–1.64), or high cumulative CPE scores (>14) (OR: 1.34, 95% CI: 1.14–1.57) had a high risk of neurological diseases. When stratified by classes of ART drugs, patients with low cumulative DDDs or low adherence had a high risk of neurological diseases, including NRTIs, PIs, NNRTIs, INSTIs, and multi-drug tablets. Subgroup analyses also suggested that patients with low cumulative DDDs or low adherence had a high risk of neurological diseases when they had high cumulative CPE scores. Patients with high cumulative DDDs or medication adherence were protected against neurological diseases only when they had low cumulative CPE scores (≤ 14). Patients may be at risk for neurological diseases when they have low cumulative DDDs, low adherence, or usage with high cumulative CPE scores. Continuous usage and low cumulative CPE scores of ART drugs may benefit neurocognitive health in patients with HIV/AIDS.

## 1 Introduction

With the introduction of combination antiretroviral therapy [cART; also known as highly active antiretroviral therapy (HAART)], HIV/AIDS has become a chronic and manageable disease (Barbier et al., 2020). Patients are advised to receive long-term cART to inhibit viral replication and control disease progression; however, HIV latency still occurs. The viral genome integrates into the human genome in circulatory immune T cells and serves as an HIV reservoir in patients treated with ART (Warren et al., 2020). Furthermore, long-term cART treatment is associated with adverse effects, including hyperlipidemia (Tsai et al., 2017a), cardiovascular disease (Dorjee et al., 2017), bone loss (Hoy et al., 2017; Chiu et al., 2021), and neurocognitive impairment (Yuan and Kaul, 2021).

Neurocognitive impairment remains an important issue in patients with HIV/AIDS (Mothobi and Brew, 2012; Yuan and Kaul, 2021); its prevalence of neurocognitive impairment varies between 15% and 65%, depending on cohort characteristics and the heterogeneity of HIV-related neurocognitive diseases (Trujillo et al., 1995; Joska et al., 2011; Dai et al., 2014). Before the cART era, neurocognitive impairment was associated with HIV virus-induced neurotoxicity and immune suppression in the central nervous system (CNS) (Brew et al., 1997; Ruhanya et al., 2022; Wallace, 2022). With cART, neurocognitive impairment became associated with loss of HIV virus replication control in patients with low ART adherence (Kamal et al., 2017). Furthermore, neurocognitive impairment is associated with ART-related neurotoxicity (Sharma, 2021), persistent low-grade chronic inflammation, immune reactivation, low-level viral replication in the CNS, and/or aging-related comorbidities (Wright et al., 2010; Shikuma et al., 2012), leading to various HIV-related neurocognitive diseases, including CNS infections, cognitive disorders, vasculopathy, and peripheral neuropathy (Tsai et al., 2017b). ART drugs are classified based on their distinct molecular mechanisms and pharmacological characteristics, such as nucleoside reverse-transcriptase inhibitors (NRTIs), protease inhibitors (PIs), non-nucleoside reverse-transcriptase inhibitors (NNRTIs), integrase strand transfer inhibitors (INSTIs), fusion inhibitors, and co-receptor antagonists (Arts and Hazuda, 2012; Nastri et al., 2023). Neurotoxicity is linked to different classes of antiretroviral drugs, including NRTIs, NNRTIs, PIs, and INSTIs (Abers et al., 2014; Xu et al., 2014; Underwood et al., 2015; Hoffmann and Llibre, 2019; Zash et al., 2019; Foster et al., 2022). Furthermore, studies have reported that NRTI administration is associated with an increased risk of neurotoxicity in both the CNS and peripheral nervous system (Blanche et al., 1999; Cepeda and Wilks, 2000). Other studies have reported that NNRTIs are associated with CNS neurotoxicity (Streck et al., 2008; Streck et al., 2011). Other studies have reported that INSTIs are associated with neuropsychiatric adverse events (Hoffmann and Llibre, 2019) and fetal neurodevelopmental abnormalities in pregnant women (Zash et al., 2019; Foster et al., 2022).

Antiretroviral drugs exhibit a variety of activities in the CNS due to blood–brain barrier (BBB) penetration (Nwogu et al., 2016). A CNS penetration effectiveness (CPE) scoring system has been developed based on each antiretroviral drug’s physiochemical properties, pharmacokinetics, and pharmacodynamics (Caniglia et al., 2014; Nwogu et al., 2016; Santos et al., 2019; Lanman et al., 2021). Antiretroviral drugs with higher CPE scores may better inhibit viral replication in the CNS and are associated with low HIV RNA levels in cerebrospinal fluid (CSF) (Marra et al., 2009; Cusini et al., 2013). However, the association between CPE scores and neurocognitive impairment remains unclear (Marra et al., 2009; Cross et al., 2013; Caniglia et al., 2014; Vassallo et al., 2014; Baker et al., 2015; Carvalhal et al., 2016). Some studies have reported that patients receiving ART with higher CPE scores are at a lower risk of neurocognitive impairment (Vassallo et al., 2014; Carvalhal et al., 2016). However, others have not found any association between CPE scores and risk of neurocognitive impairment (Cross et al., 2013; Baker et al., 2015) or even an opposite association (Marra et al., 2009; Caniglia et al., 2014).

In this nested case-control study, we explored the association between ART exposure and the risk of neurological diseases (CNS infections, cognitive disorders, vasculopathy, and peripheral neuropathy) in patients with HIV/AIDS in Taiwan. In addition, detailed associations between ART usage, timing of exposure, cumulative DDD, adherence, cumulative CPE score, and risk of neurological diseases were investigated.

## 2 Materials and methods

### 2.1 Approvals

This study was approved by the China Medical University Hospital (CMUH) in Taichung, Taiwan [Approval number: CMUH107-REC3-074 (CR1)]. Individuals were analyzed anonymously in the database, and informed consent was not required.

### 2.2 Study population

**FIGURE 1:**
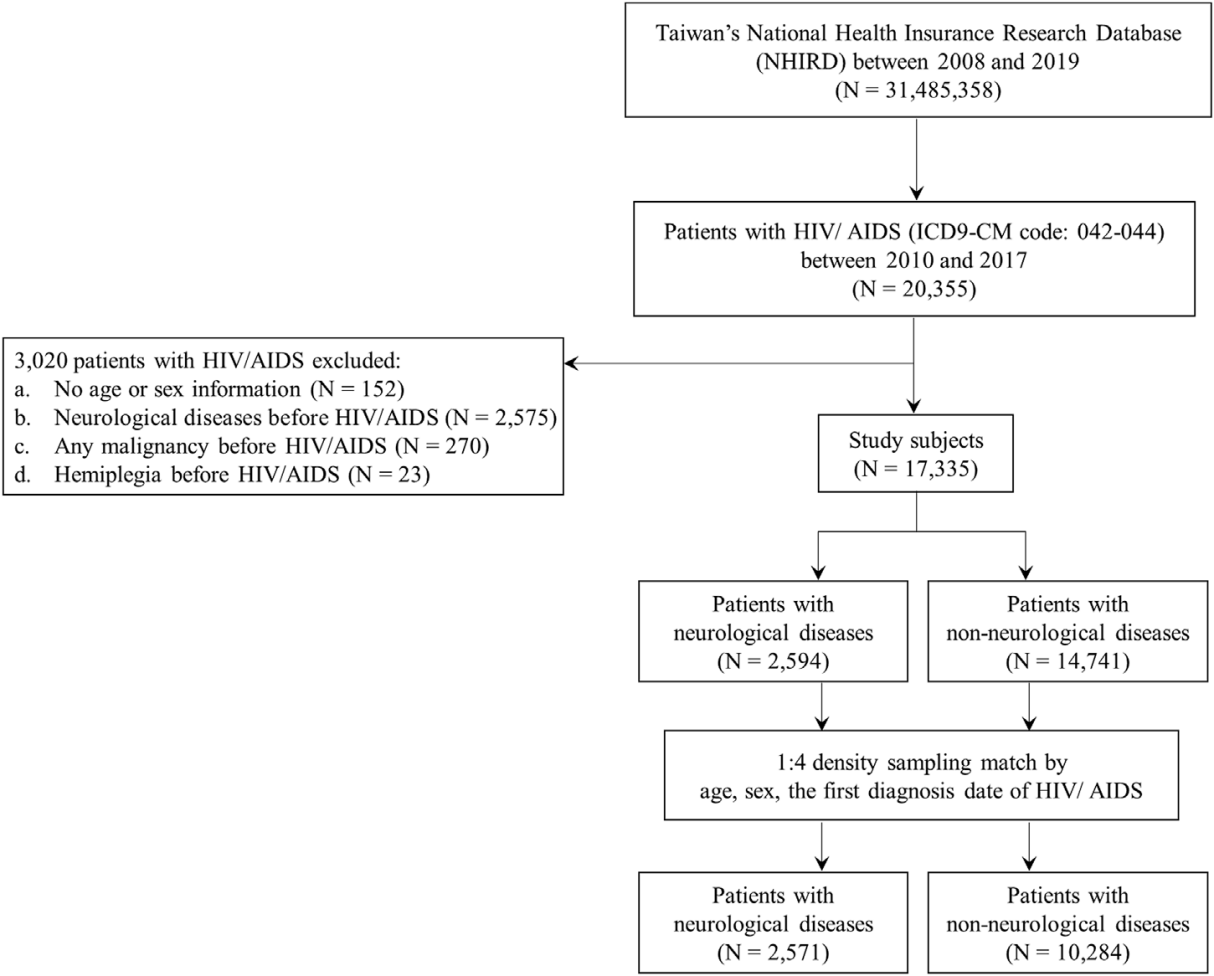
Flow chart for the recruitment of study subjects. Inclusion and exclusion criteria for neurological disease cases and non-neurological disease controls in patients with HIV/AIDS. HIV, human immunodeficiency virus; AIDS, acquired immune deficiency syndrome.

We identified 20,355 patients with HIV/AIDS between 2010 and 2017 from the National Health Insurance Research Database (NHIRD). HIV/AIDS was determined by at least one inpatient or three outpatient visits within 1 year using the International Classification of Diseases, Ninth Revision, Clinical Modification (ICD-9-CM codes):042–044 (Figure 1). After applying the following exclusion criteria, 17,335 patients were included: 1) patients with no age or sex information (*N* = 152); 2) patients with neurological disease before HIV/AIDS (*N* = 2,575); 3) patients with any malignancy before HIV/AIDS (*N* = 270); and 4) patients with hemiplegia before HIV/AIDS (*N* = 23). Neurological disease incidences were determined by at least one inpatient or three outpatient visits within 1 year using the ICD-9-CM codes: 1) CNS infections (ICD-9-CM codes: 013, 047, 053, 094, 200, 320, 321, 322, 323, 003.21, 054.3, 054.4, 098.82, 112.83, 114.2, 115.91, 130.0, and 321.0); 2) cognitive disorders (ICD-9-CM codes: 290, 293, 294, 332, 345, 348.1, 348.3, and 780.3); 3) vasculopathy (ICD-9-CM codes: 325, 430, 431, 432, 433, 434, 435, 436, and 437); and 4) peripheral neuropathy (ICD-9-CM codes: 350, 351, 353, 354, 355, 356, 357, and 358) (Tsai et al., 2017b). Hemiplegia was determined using the ICD-9-CM codes: 334.1, 342, 343, 344.9, and 344.0–344.6. Malignancy was determined using the ICD-9-CM codes: 140–208. More than 75% of inpatient and outpatient visits were conducted in the clinics, including infectious diseases, international medicine, emergency medicine, and psychiatry (Supplementary Table S1). Furthermore, according to the frequency distribution of neurological disease subtypes among patients with neurological diseases, more than 64% of patients had CNS infections (1,681 out of the 2,594 patients with neurological diseases) (Supplementary Table S2). Among patients with HIV/AIDS, more than 9% of patients had CNS infections (1,684 out of a total of 17,335 subjects) (Supplementary Table S2).

### 2.3 Cases and controls

**FIGURE 2:**
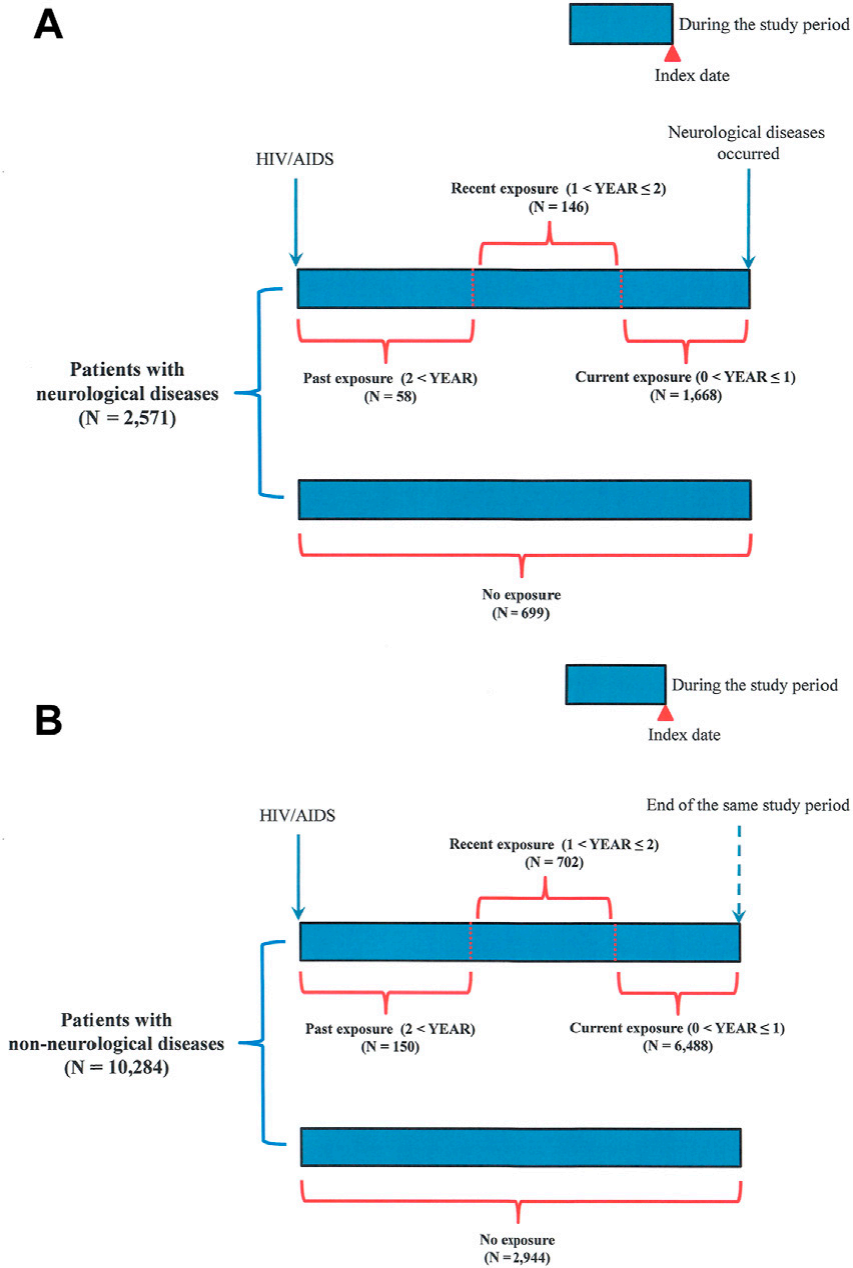
Follow-up time in patients with HIV/AIDS. **(A)** Patients with neurological diseases; **(B)** patients with non-neurological diseases. Past, recent, and current exposures to ART drugs were defined according to the latest ART prescription date before the index date. The date of the first neurological disease diagnosis was designated as the index date. Patients who received ART drugs during the time window of over 2 years before the index date but were currently non-users were defined as patients with past exposure to ART drugs. Patients who received ART drugs within the time window of 1–2 years before the index date were defined as patients with recent exposure to ART drugs. Patients who received ART drugs within the time window of 0–1 year before the index date were defined as patients with current exposure to ART drugs. Patients who continuously received ART since their first diagnosis of HIV/AIDS were also defined as those with current exposure to ART drugs.

The cases in this nested case-control study were defined as patients with HIV/AIDS who were first diagnosed with neurological diseases during the study period (*N* = 2,594) (Figures 1, 2). The study period was defined as the period between the date of the first diagnosis of HIV/AIDS and that of the first diagnosis of neurological diseases. The index date was designated as the date of the first diagnosis of neurological disease. Patients with HIV/AIDS who were not diagnosed with neurological diseases during the study period were defined as controls (N = 14,741) (Figures 1, 2). To avoid potential confounding factors, the density-sampling matching approach was used to match neurological and non-neurological disease groups in a ratio of 1:4 for cases and controls using sex, age, and the date of the first diagnosis of HIV/AIDS. After matching the cases and controls, 2,571 neurological disease cases and 10,284 non-neurological disease controls were included in the study (Figures 1, 2).

### 2.4 Exposure to antiretroviral therapy (ART) drugs

This study included the following antiretroviral therapy (ART) drugs: 1) nucleoside/nucleotide reverse-transcriptase inhibitor (NRTI), Anatomical Therapeutic Chemical (ATC) code: J05AF01, J05AF04, J05AF02, J05AF03, J05AF05, J05AF06, and J05AF07; 2) non-nucleoside reverse-transcriptase inhibitor (NNRTI), ATC code: J05AG01, J05AG04, J05AG03, and J05AG05; 3) protease inhibitor (PI), ATC code: J05AE01, J05AE04, J05AE02, J05AE03, J05AE08, J05AE09, and J05AE10; 4) integrase strand transfer inhibitor (INSTI), ATC code: J05AX09, J05AX07, J05AX08, and J05AX12; and 5) combinations of over two ART drugs per tablet, ATC code: J05AR01, J05AR03, J05AR02, J05AR04, J05AR05, J05AR06, J05AR08, J05AR10, J05AR13, J05AR14, J05AR18, J05AR19, J05AR20, J05AR21, J05AR22, and J05AR25 (Chiu et al., 2021).

ART use was defined as receiving ART during the study period (Table 2). Non-ART use was defined as the absence of ART prescriptions during the study period. Regarding the timing of ART drug exposure, three categories were defined according to the latest ART prescription date before the index date (Figure 2; Table 2). The date of the first diagnosis of neurological disease was designated as the index date. The three categories included past exposure (2 < YEAR), recent exposure (1 < YEAR ≤ 2), and current exposure (0 < YEAR ≤ 1), according to previous similar studies (Weng et al., 2019). Patients who received ART drugs during the time window of over 2 years before the index date but were currently non-users were defined as patients with past exposure (Figure 2). Patients who received ART drugs within the time window of 1–2 years before the index date were defined as patients with recent exposure (Figure 2). Patients who received ART drugs within the time window of 0–1 year before the index date were defined as patients with current exposure (Figure 2). Patients who continuously received ART since their first diagnosis of HIV/AIDS were also defined as those with current exposure (Figure 2).

Defined daily doses (DDDs) were recommended by the Collaborating Center for Drug Statistics Methodology of the World Health Organization (WHO) (https://www.whocc.no/atc_ddd_index/). For the cumulative DDD of ART drugs, two categorized cumulative DDDs were defined as (total amount of drug)/(amount of drug in a DDD) from the first date of ART treatment to the index date, according to previous studies (Wang et al., 2019). The two categories of cumulative DDDs included cumulative DDDs ≥ 2,500 and cumulative DDDs < 2,500 (Table 2). For adherence (ADH) (electronic prescription claim data adherence) to ART drugs, two categorized ADHs were defined according to (total number of prescribed days)/(total number of observation days) from the first date of ART treatment to the index date, according to previous studies (Grymonpre et al., 1998). The two categorized ADHs included high-adherence (0.8 < ADH) and low-adherence (0 < adherence (ADH) ≤ 0.8) groups (Table 2). For the central nervous system penetration effectiveness (CPE), four categorized CPE scores were defined according to the sum of the ranks of each ART drug in the regimen administered to a patient from the start of drug use to the first diagnosis date of neurological diseases (Table 2). The rank of each ART drug was determined based on previous studies (Caniglia et al., 2014; Santos et al., 2019; Lanman et al., 2021; Yuan and Kaul, 2021). The CPE score for a particular regimen was determined by adding the rankings for each ART drug included in the regimen. If the regimen was altered, the ranking of the newly added ART drug was added to the CPE score accumulated during the study period. We designed a population-based nested case-control study similar to that of previous reports (Daneman et al., 2021), and the cumulative CPE score was estimated over the study period (follow-up years, between the date of the first HIV/AIDS diagnosis and the date of the occurrence of neurological diseases). Both the cases and controls were followed-up for the same period of time. The four CPE score categories included the 0 ≤ CPE score ≤ 10, 10 < CPE score ≤ 14, 14 < CPE score ≤ 18, and 18 < CPE score groups. The cumulative DDD, adherence, and CPE scores were estimated during the study period.

### 2.5 Data collection

The patients were selected from inpatient and outpatient records based on age, sex, diagnosis, admission, procedures, and prescriptions. Demographic characteristics, including sex, age, Charlson comorbidity index (CCI) score, ART usage, and follow-up period for patients with HIV/AIDS were collected (Tables 1, 2). Charlson comorbidity index (CCI) scores were determined within the first date of the HIV/AIDS diagnosis.

**TABLE 1:** Demographic characteristics of patients with HIV/AIDS in Taiwan (total subjects and density-sampling matched subjects).

Characteristics	Total subjects		*p*-value	Matched subjects		*p*-value
	Patients with neurological diseases	Patients with non-neurological diseases		Patients with neurological diseases	Patients with non-neurological diseases	
	(*N* = 2,594)	(*N* = 14,741)		(*N* = 2,571)	(*N* = 10,284)	
	*N* (%)	*N* (%)		*N* (%)	*N* (%)	
Follow-up years (median (IQR))	1.98 (3.52)	5.38 (4.14)	***<0.001***	1.97 (3.53)	1.97 (3.53)	1
Age (median (IQR))	31.96 (13.46)	29.26 (11.38)	***<0.001***	31.88 (13.18)	31.89 (13.16)	0.376
Sex			***0.015***			1
Male	2,467 (95.10%)	14,169 (96.12%)		2,456 (95.53%)	9,824 (95.53%)	
Female	127 (4.90%)	572 (3.88%)		115 (4.47%)	460 (4.47%)	
Charlson comorbidity number			***0.005***			***0.022***
0	2022 (77.95%)	11,888 (80.65%)		2011 (78.22%)	8,274 (80.46%)	
1–2	547 (21.09%)	2,746 (18.63%)		536 (20.85%)	1943 (18.89%)	
≥ 3	25 (0.96%)	107 (0.73%)		24 (0.93%)	67 (0.65%)	
ART usage			***< 0.001***			0.148
Non-ART use	702 (27.06%)	893 (6.06%)		699 (27.19%)	2,944 (28.63%)	
ART use	1892 (72.94%)	13,848 (93.94%)		1872 (72.81%)	7,340 (71.37%)	

N, number; ART, antiretroviral therapy; SD, standard deviation; HIV, human immunodeficiency virus; AIDS, acquired immune deficiency syndrome. The density-sampling matching method was performed using age, sex, and the first diagnosis date of HIV/AIDS. Age, sex, and comorbidities were identified within the HIV/AIDS diagnosis date. Follow-up years and ART usage were identified during the period between HIV/AIDS and neurological diseases. Significant *p*-values (*p* < 0.05) are highlighted in bold italic font.

**TABLE 2:** Odds ratios for neurological diseases when exposed to ART drugs.

Characteristics	Patients with neurological diseases	Patients with non-neurological diseases	Crude			Adjusted		
	(*N* = 2,571)	(*N* = 10,284)						
	*N* (%)	*N* (%)	OR	95% CI	*p*-value	OR	95% CI	*p*-value
ART usage								
Non-ART use	699 (27.19%)	2,944 (28.63%)	1	Ref	Ref	1	Ref	Ref
ART use	1872 (72.81%)	7,340 (71.37%)	1.09	(0.98–1.21)	0.117	1.08	(0.97–1.2)	0.148
Timing of exposure to ART drugs								
Non-ART use	699 (27.19%)	2,944 (28.63%)	1	Ref	Ref	1	Ref	Ref
Current exposure (0 < YEAR ≤ 1)	1,668 (64.88%)	6,488 (63.09%)	1.09	(0.98–1.22)	0.094	1.09	(0.98–1.21)	0.122
Recent exposure (1 < YEAR ≤ 2)	146 (5.68%)	702 (6.83%)	0.88	(0.71–1.08)	0.229	0.88	(0.71–1.08)	0.224
Past exposure (2 < YEAR)	58 (2.26%)	150 (1.46%)	1.67	(1.21–2.30)	***0.002***	1.68	(1.22–2.32)	***0.002***
Cumulative defined daily dose (DDD) of ART drugs								
Non-ART use	699 (27.19%)	2,944 (28.63%)	1	Ref	Ref	1	Ref	Ref
Cumulative DDDs < 2,500	1,448 (56.32%)	4,879 (47.44%)	1.29	(1.15–1.43)	***<0.001***	1.28	(1.15–1.42)	***<0.001***
Cumulative DDDs ≥ 2,500	424 (16.49%)	2,461 (23.93%)	0.54	(0.46–0.63)	***<0.001***	0.53	(0.45–0.62)	***<0.001***
Adherence (ADH) of ART drugs								
Non-ART use	699 (27.19%)	2,944 (28.63%)	1	Ref	Ref	1	Ref	Ref
Low (0 < ADH ≤ 0.8)	1,054 (41.00%)	3,067 (29.82%)	1.47	(1.31–1.64)	***<0.001***	1.46	(1.30–1.64)	***<0.001***
High (0.8 < ADH)	818 (31.82%)	4,273 (41.55%)	0.79	(0.70–0.90)	***<0.001***	0.78	(0.70–0.88)	***<0.001***
Cumulative CPE scores of ART drugs								
Non-ART use	699 (27.19%)	2,944 (28.63%)	1	Ref	Ref	1	Ref	Ref
0 ≦ Cumulative CPE score ≦ 10	911 (35.43%)	3,770 (36.66%)	1.03	(0.92–1.16)	0.576	1.03	(0.92–1.15)	0.632
10 < Cumulative CPE score ≦14	403 (15.67%)	1716 (16.69%)	1.03	(0.89–1.19)	0.694	1.02	(0.88–1.18)	0.785
14 < Cumulative CPE score ≦ 18	337 (13.11%)	1,106 (10.75%)	1.35	(1.15–1.58)	***<0.001***	1.34	(1.14–1.57)	***<0.001***
18 < Cumulative CPE score	221 (8.60%)	748 (7.27%)	1.34	(1.11–1.62)	***0.002***	1.33	(1.10–1.61)	***0.003***

Follow-up years (mean ± SD) (the period between the HIV/AIDS diagnosis date and the neurological disease diagnosis date): 2.62 ± 2.31 years. N, number; ART, antiretroviral therapy; DDD, defined daily dose; ADH, adherence of ART drugs; CPE score, central nervous system penetration effectiveness (CPE) score; OR, odds ratio; CI, confidence interval; Ref, reference. Model was applied using conditional logistic regression analysis adjusted by Charlson comorbidity number. Comorbidities were identified within the HIV/AIDS diagnosis date. Cumulative defined daily dose (DDD), adherence (ADH), and central nervous system penetration effectiveness (CPE) score were calculated during the period between the HIV/AIDS diagnosis date and the neurological disease diagnosis date. The date of the first diagnosis of neurological disease was designated as the index date. The three categories included past exposure (2 < YEAR), recent exposure (1 < YEAR ≤ 2), and current exposure (0 < YEAR ≤ 1) according to the previous similar studies (PubMed PMID number: 31374345). Patients who received ART drugs during the time window of over 2 years before the index date but were currently non-users were defined as patients with past exposure. Patients who received ART drugs during the time window of 1–2 years before the index date were defined as patients with recent exposure. Patients who received ART drugs during the time window of 0–1 year before the index date were defined as patients with current exposure. Patients who had continuously received ART since their first diagnosis of HIV/AIDS were also defined as those with current exposure. The defined daily doses (DDDs) were those recommended by the Collaborating Center for Drug Statistics Methodology of the World Health Organization (WHO) (https://www.whocc.no/atc_ddd_index/). For the cumulative defined daily dose (DDD) of ART drugs, two categorized cumulative DDDs were defined according to (total amount of drug)/(amount of drug in a DDD) from the first date of ART treatment to the index date according to previous studies (PubMed PMID number: 31888519). For adherence (ADH) (electronic prescription claim data adherence) to ART drugs, two categorized ADHs were defined according to (total number of prescribed days)/(total number of observation days) from the first date of ART treatment to the index date according to previous studies (PubMed PMID number: 9681089). For the central nervous system penetration effectiveness (CPE) score, four categorized CPE scores were defined according to the sum of the ranks of each ART drug in the regimen administered to a patient from the start of drug usage to the first diagnosis date of neurological diseases (Table 2). The rank of each ART drug was determined according to previous studies (PubMed PMID number: 24907236, 31304188, 31823251, and 31385157). The CPE score for a particular regimen was determined by adding together the rankings of each ART drug included in the regimen. If the regimen was altered, the ranking of the newly added ART drug was added to the accumulated CPE score during the study period. Significant *p*-values (*p* < 0.05) are highlighted in bold italic font.

### 2.6 Statistical analyses

**FIGURE 3:**
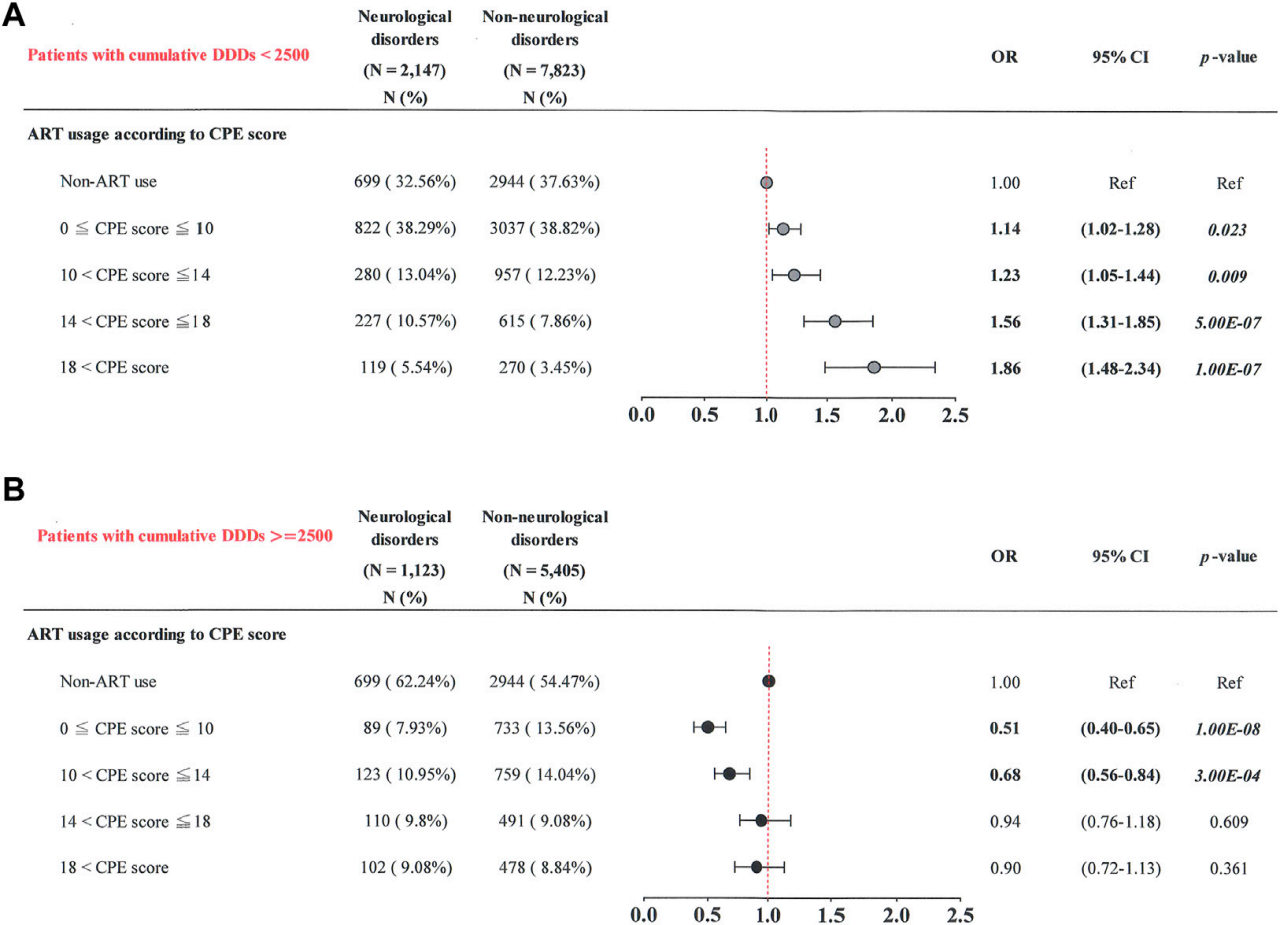
Cumulative CPE score and risk of neurological diseases when stratified by cumulative DDD of ART. **(A)** Patients with cumulative DDDs < 2,500; **(B)** patients with cumulative DDDs ≥ 2,500.

**FIGURE 4:**
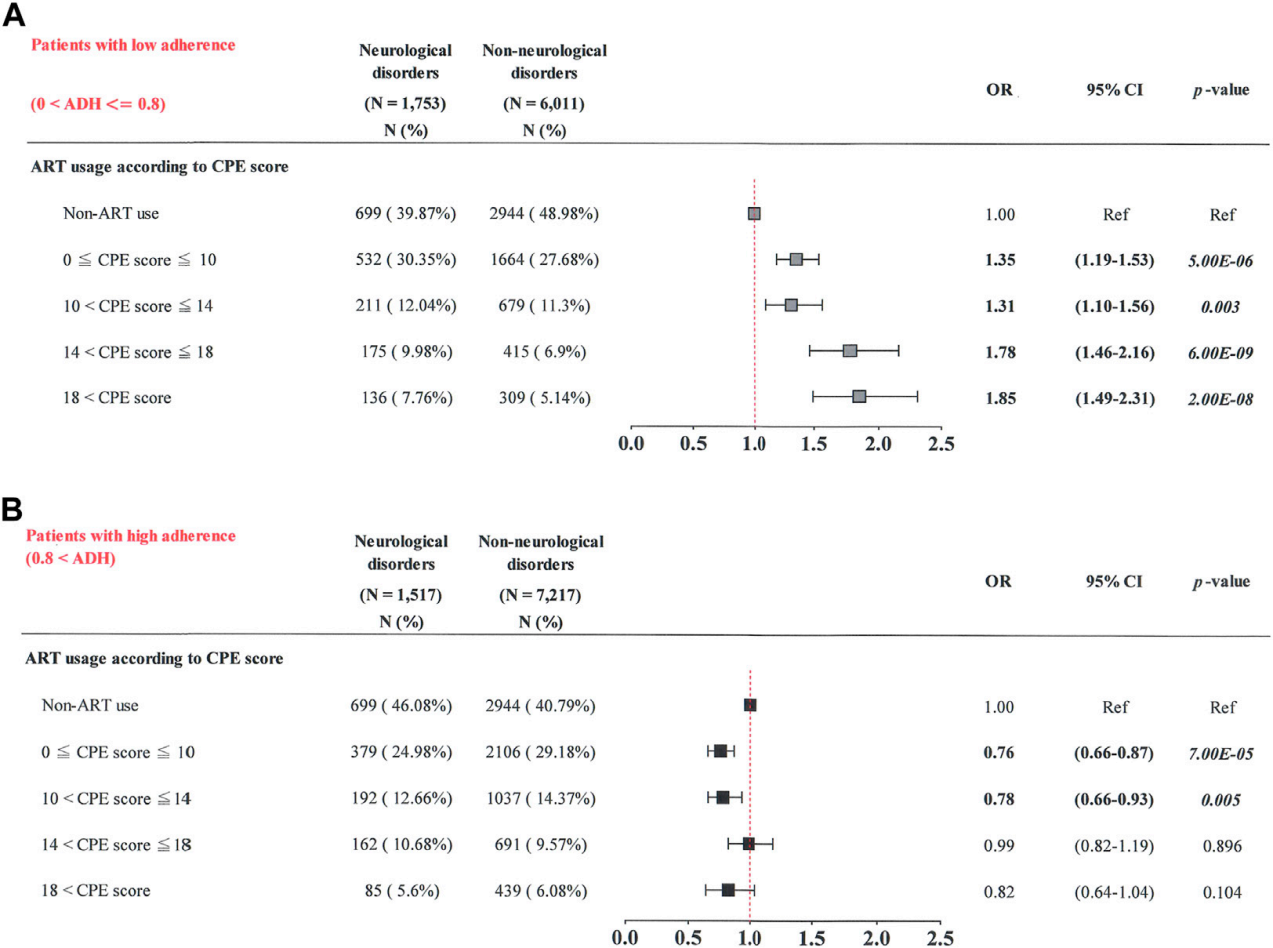
Cumulative CPE score of ART drugs and risk of neurological diseases when stratified by adherence (ADH) to ART drugs. **(A)** Patients with low adherence (0 < ADH ≤ 0.8); **(B)** patients with high adherence (0.8 < ADH).

Chi-square tests were performed to compare categorical variables (sex, Charlson comorbidity number, ART usage, substance use disorders, HBV, HCV, and syphilis infections) between the neurological disease cases and non-neurological disease controls (Table 1) (Supplementary Table S5). The Wilcoxon rank-sum test was used to compare continuous variables (age and follow-up years) between the neurological disease cases and non-neurological disease controls (Table 1). A multivariate conditional logistic regression model was used to evaluate the association between ART and the risk of neurological diseases, adjusted by the Charlson comorbidity number (Table 2) (Supplementary Tables S3, S4) (Tables 3–7). Subgroup analysis and conditional logistic regression model according to cumulative DDDs were used to estimate the association between cumulative CPE score and the risk of neurological diseases (Figure 3A: patients with cumulative DDDs < 2,500; Figure 3B: patients with cumulative DDDs ≥ 2,500). Subgroup analysis and conditional logistic regression model according to adherence were used to estimate the association between cumulative CPE score and risk of neurological diseases [Figure 4A: patients with low adherence (0 < ADH ≤ 0.8); Figure 4B: patients with high adherence (0.8 < ADH)]. Odds ratios (ORs) and 95% confidence intervals (CIs) were also calculated. Statistical Analysis System (SAS) software (version 9.3; SAS Institute, Cary, NC, United States) was used to manage and analyze the data. Statistical significance was set at *p* < 0.05.

**TABLE 3:** Odds ratios for neurological diseases when exposed to NRTI drugs.

Characteristics	Patients with neurological diseases	Patients with non-neurological diseases	Crude			Adjusted		
	(*N* = 2,571)	(*N* = 10,284)						
	*N* (%)	*N* (%)	OR	95% CI	*p*-value	OR	95% CI	*p*-value
NRTI usage								
Non-exposure	2050 (79.74%)	8,302 (80.73%)	1	Ref	Ref	1	Ref	Ref
Exposure	521 (20.26%)	1982 (19.27%)	1.07	(0.96–1.20)	0.238	1.06	(0.95–1.19)	0.291
Timing of exposure to NRTI drugs								
Non-exposure	2050 (79.74%)	8,302 (80.73%)	1	Ref	Ref	1	Ref	Ref
Current exposure (0 < YEAR ≤ 1)	288 (11.2%)	938 (9.12%)	1.27	(1.10–1.47)	***0.001***	1.26	(1.09–1.46)	***0.002***
Recent exposure (1 < YEAR ≤ 2)	80 (3.11%)	364 (3.54%)	0.88	(0.68–1.13)	0.319	0.87	(0.68–1.12)	0.286
Past exposure (2 < YEAR)	153 (5.95%)	680 (6.61%)	0.87	(0.72–1.07)	0.188	0.87	(0.71–1.06)	0.172
Cumulative defined daily dose (DDD) of NRTI drugs								
Non-exposure	2050 (79.74%)	8,302 (80.73%)	1	Ref	Ref	1	Ref	Ref
Cumulative DDDs < 564	329 (12.8%)	924 (8.98%)	1.47	(1.28–1.69)	***<0.001***	1.46	(1.27–1.68)	***<0.001***
Cumulative DDDs ≥ 564	192 (7.47%)	1,058 (10.29%)	0.71	(0.60–0.84)	***<0.001***	0.70	(0.60–0.83)	***<0.001***
Adherence (ADH) of NRTI drugs								
Non-exposure	2050 (79.74%)	8,302 (80.73%)	1	Ref	Ref	1	Ref	Ref
Low (0 < ADH ≤ 0.8)	434 (16.88%)	1,578 (15.34%)	1.12	(0.99–1.27)	0.060	1.12	(0.99–1.26)	0.075
High (0.8 < ADH)	87 (3.38%)	404 (3.93%)	0.87	(0.68–1.11)	0.254	0.86	(0.68–1.09)	0.220

Follow-up years (mean ± SD) (the period between the HIV/AIDS diagnosis date and the neurological disease diagnosis date): 2.62 ± 2.31 years. N, number; NRTI, nucleoside reverse-transcriptase inhibitors; DDD, defined daily dose; ADH, adherence of ART drugs; OR, odds ratio; CI, confidence interval; Ref, reference. Model was applied using conditional logistic regression analysis adjusted by Charlson comorbidity number. Comorbidities were identified within the HIV/AIDS diagnosis date. Cumulative defined daily dose (DDD) and adherence (ADH) were calculated during the period between the HIV/AIDS diagnosis date and the neurological disease diagnosis date. The date of the first diagnosis of neurological disease was designated as the index date. The three categories included past exposure (2 < YEAR), recent exposure (1 < YEAR ≤ 2), and current exposure (0 < YEAR ≤ 1) according to the previous similar studies (PubMed PMID number: 31374345). Patients who received ART drugs during the time window of over 2 years before the index date but were currently non-users were defined as patients with past exposure. Patients who received ART drugs during the time window of 1–2 years before the index date were defined as patients with recent exposure. Patients who received ART drugs during the time window of 0–1 year before the index date were defined as patients with current exposure. Patients who had continuously received ART since their first diagnosis of HIV/AIDS were also defined as those with current exposure. The defined daily doses (DDDs) were those recommended by the Collaborating Center for Drug Statistics Methodology of the World Health Organization (WHO) (https://www.whocc.no/atc_ddd_index/). For the cumulative defined daily dose (DDD) of ART drugs, two categorized cumulative DDDs were defined according to (total amount of drug)/(amount of drug in a DDD) from the first date of ART treatment to the index date according to previous studies (PubMed PMID number: 31888519). For adherence (ADH) (electronic prescription claim data adherence) to ART drugs, two categorized ADHs were defined according to (total number of prescribed days)/(total number of observation days) from the first date of ART treatment to the index date according to previous studies (PubMed PMID number: 9681089). Significant *p*-values (*p* < 0.05) are highlighted in bold italic font.

**TABLE 4:** Odds ratios for neurological diseases when exposed to PI drugs.

Characteristics	Patients with neurological diseases	Patients with non-neurological diseases	Crude			Adjusted		
	(*N* = 2,571)	(*N* = 10,284)						
	*N* (%)	*N* (%)	OR	95% CI	*p*-value	OR	95% CI	*p*-value
PI usage								
Non-exposure	2,223 (86.46%)	8,987 (87.39%)	1	Ref	Ref	1	Ref	Ref
Exposure	348 (13.54%)	1,297 (12.61%)	1.09	(0.96–1.24)	0.200	1.09	(0.95–1.24)	0.219
Timing of exposure to PI drugs								
Non-exposure	2,223 (86.46%)	8,987 (87.39%)	1	Ref	Ref	1	Ref	Ref
Current exposure (0 < YEAR ≤ 1)	241 (9.37%)	813 (7.91%)	1.20	(1.03–1.40)	***0.019***	1.20	(1.03–1.39)	***0.021***
Recent exposure (1 < YEAR ≤ 2)	43 (1.67%)	216 (2.1%)	0.80	(0.58–1.12)	0.200	0.80	(0.57–1.11)	0.184
Past exposure (2 < YEAR)	64 (2.49%)	268 (2.61%)	0.96	(0.72–1.28)	0.788	0.96	(0.72–1.28)	0.788
Cumulative defined daily dose (DDD) of PI drugs								
Non-exposure	2,223 (86.46%)	8,987 (87.39%)	1	Ref	Ref	1	Ref	Ref
Cumulative DDDs < 411	212 (8.25%)	611 (5.94%)	1.41	(1.20–1.66)	***<0.001***	1.41	(1.19–1.66)	***<0.001***
Cumulative DDDs ≥ 411	136 (5.29%)	686 (6.67%)	0.79	(0.65–0.96)	***0.018***	0.78	(0.64–0.95)	***0.015***
Adherence (ADH) of PI drugs								
Non-exposure	2,223 (86.46%)	8,987 (87.39%)	1	Ref	Ref	1	Ref	Ref
Low (0 < ADH ≤ 0.8)	290 (11.28%)	914 (8.89%)	1.29	(1.12–1.49)	***<0.001***	1.29	(1.12–1.49)	***<0.001***
High (0.8 < ADH)	58 (2.26%)	383 (3.72%)	0.61	(0.46–0.81)	***<0.001***	0.60	(0.46–0.80)	***<0.001***

Follow-up years (mean ± SD) (the period between the HIV/AIDS diagnosis date and the neurological disease diagnosis date): 2.62 ± 2.31 years. N, number; PI, protease inhibitors; DDD, defined daily dose; ADH, adherence of ART drugs; OR, odds ratio; CI, confidence interval; Ref, reference. Model was applied using conditional logistic regression analysis adjusted by Charlson comorbidity number. Comorbidities were identified within the HIV/AIDS diagnosis date. Cumulative defined daily dose (DDD) and adherence (ADH) were calculated during the period between the HIV/AIDS diagnosis date and the neurological disease diagnosis date. The date of the first diagnosis of neurological disease was designated as the index date. The three categories included past exposure (2 < YEAR), recent exposure (1 < YEAR ≤ 2), and current exposure (0 < YEAR ≤ 1) according to the previous similar studies (PubMed PMID number: 31374345). Patients who received ART drugs during the time window of over 2 years before the index date but were currently non-users were defined as patients with past exposure. Patients who received ART drugs during the time window of 1–2 years before the index date were defined as patients with recent exposure. Patients who received ART drugs during the time window of 0–1 year before the index date were defined as patients with current exposure. Patients who had continuously received ART since their first diagnosis of HIV/AIDS were also defined as those with current exposure. The defined daily doses (DDDs) were those recommended by the Collaborating Center for Drug Statistics Methodology of the World Health Organization (WHO) (https://www.whocc.no/atc_ddd_index/). For the cumulative defined daily dose (DDD) of ART drugs, two categorized cumulative DDDs were defined according to (total amount of drug)/(amount of drug in a DDD) from the first date of ART treatment to the index date according to previous studies (PubMed PMID number: 31888519). For adherence (ADH) (electronic prescription claims data adherence) to ART drugs, two categorized ADHs were defined according to (total number of prescribed days)/(total number of observation days) from the first date of ART treatment to the index date according to previous studies (PubMed PMID number: 9681089). Significant *p*-values (*p* < 0.05) are highlighted in bold italic font.

**TABLE 5:** Odds ratios for neurological diseases when exposed to NNRTI drugs.

Characteristics	Patients with neurological diseases	Patients with non-neurological diseases	Crude			Adjusted		
	(*N* = 2,571)	(*N* = 10,284)						
	*N* (%)	*N* (%)	OR	95% CI	*p*-value	OR	95% CI	*p*-value
NNRTI usage								
Non-exposure	1,228 (47.76%)	5,092 (49.51%)	1	Ref	Ref	1	Ref	Ref
Exposure	1,343 (52.24%)	5,192 (50.49%)	1.08	(0.99–1.19)	0.087	1.08	(0.99–1.19)	0.099
Timing of exposure to NNRTI drugs								
Non-exposure	1,228 (47.76%)	5,092 (49.51%)	1	Ref	Ref	1	Ref	Ref
Current exposure (0 < YEAR ≤ 1)	767 (29.83%)	2,836 (27.58%)	1.13	(1.02–1.26)	***0.020***	1.13	(1.02–1.26)	***0.024***
Recent exposure (1 < YEAR ≤ 2)	205 (7.97%)	867 (8.43%)	0.98	(0.82–1.17)	0.819	0.98	(0.82–1.16)	0.806
Past exposure (2 < YEAR)	371 (14.43%)	1,489 (14.48%)	1.02	(0.88–1.20)	0.765	1.02	(0.87–1.19)	0.786
Cumulative defined daily dose (DDD) of NNRTI drugs								
Non-exposure	1,228 (47.76%)	5,092 (49.51%)	1	Ref	Ref	1	Ref	Ref
Cumulative DDDs < 226	802 (31.19%)	2,470 (24.02%)	1.37	(1.23–1.52)	***<0.001***	1.37	(1.23–1.52)	***<0.001***
Cumulative DDDs ≥ 226	541 (21.04%)	2,722 (26.47%)	0.78	(0.69–0.88)	***<0.001***	0.78	(0.69–0.88)	***<0.001***
Adherence (ADH) of NNRTI drugs								
Non-exposure	1,228 (47.76%)	5,092 (49.51%)	1	Ref	Ref	1	Ref	Ref
Low (0 < ADH ≤ 0.8)	1,047 (40.72%)	3,761 (36.57%)	1.18	(1.07–1.30)	***0.001***	1.18	(1.06–1.30)	***0.001***
High (0.8 < ADH)	296 (11.51%)	1,431 (13.91%)	0.86	(0.74–0.99)	***0.041***	0.86	(0.74–0.99)	***0.034***

Follow-up years (mean ± SD) (the period between the HIV/AIDS diagnosis date and the neurological disease diagnosis date ): 2.62 ± 2.31 years. N, number; NNRTI, non-nucleoside reverse-transcriptase inhibitors; DDD, defined daily dose; ADH, adherence of ART, drugs; OR, odds ratio; CI, confidence interval; Ref, reference. Model was applied using conditional logistic regression analysis adjusted by Charlson comorbidity number. Comorbidities were identified within the HIV/AIDS diagnosis date. Cumulative defined daily dose (DDD) and adherence (ADH) were calculated during the period between the HIV/AIDS diagnosis date and the neurological disease diagnosis date. The date of the first diagnosis of neurological disease was designated as the index date. The three categories included past exposure (2 < YEAR), recent exposure (1 < YEAR ≤ 2), and current exposure (0 < YEAR ≤ 1) according to the previous similar studies (PubMed PMID number: 31374345). Patients who received ART drugs during the time window of over 2 years before the index date but were currently non-users were defined as patients with past exposure. Patients who received ART drugs during the time window of 1–2 years before the index date were defined as patients with recent exposure. Patients who received ART drugs during the time window of 0–1 year before the index date were defined as patients with current exposure. Patients who had continuously received ART since their first diagnosis of HIV/AIDS were also defined as those with current exposure. The defined daily doses (DDDs) were those recommended by the Collaborating Center for Drug Statistics Methodology of the World Health Organization (WHO) (https://www.whocc.no/atc_ddd_index/). For the cumulative defined daily dose (DDD) of ART drugs, two categorized cumulative DDDs were defined according to (total amount of drug)/(amount of drug in a DDD) from the first date of ART treatment to the index date according to previous studies (PubMed PMID number: 31888519). For adherence (ADH) (electronic prescription claims data adherence) to ART drugs, two categorized ADHs were defined according to (total number of prescribed days)/(total number of observation days) from the first date of ART treatment to the index date according to previous studies (PubMed PMID number: 9681089). Significant *p*-values (*p* < 0.05) are highlighted in bold italic font.

**TABLE 6:** Odds ratios for neurological diseases when exposed to INSTI drugs.

Characteristics	Patients with neurological diseases	Patients with non-neurological diseases	Crude			Adjusted		
	(*N* = 2,571)	(*N* = 10,284)						
	*N* (%)	*N* (%)	OR	95% CI	*p*-value	OR	95% CI	*p*-value
INSTI usage								
Non-exposure	2,341 (91.05%)	9,530 (92.67%)	1	Ref	Ref	1	Ref	Ref
Exposure	230 (8.95%)	754 (7.33%)	1.25	(1.07–1.47)	***0.005***	1.25	(1.06–1.46)	***0.007***
Timing of exposure to INSTI drugs								
Non-exposure	2,341 (91.05%)	9,530 (92.67%)	1	Ref	Ref	1	Ref	Ref
Current exposure (0 < YEAR ≤ 1)	156 (6.07%)	461 (4.48%)	1.39	(1.15–1.68)	***<0.001***	1.38	(1.14–1.66)	***<0.001***
Recent exposure (1 < YEAR ≤ 2)	42 (1.63%)	152 (1.48%)	1.14	(0.80–1.61)	0.471	1.13	(0.80–1.60)	0.491
Past exposure (2 < YEAR)	32 (1.24%)	141 (1.37%)	0.93	(0.63–1.37)	0.712	0.93	(0.63–1.37)	0.702
Cumulative defined daily dose (DDD) of INSTI drugs								
Non-exposure	2,341 (91.05%)	9,530 (92.67%)	1	Ref	Ref	1	Ref	Ref
Cumulative DDDs < 305	142 (5.52%)	351 (3.41%)	1.66	(1.35–2.03)	***<0.001***	1.64	(1.34–2.01)	***<0.001***
Cumulative DDDs ≥ 305	88 (3.42%)	403 (3.92%)	0.89	(0.70–1.13)	0.339	0.89	(0.70–1.13)	0.323
Adherence (ADH) of INSTI drugs								
Non-exposure	2,341 (91.05%)	9,530 (92.67%)	1	Ref	Ref	1	Ref	Ref
Low (0 < ADH ≤ 0.8)	207 (8.05%)	609 (5.92%)	1.39	(1.18–1.64)	***<0.001***	1.38	(1.17–1.63)	***<0.001***
High (0.8 < ADH)	23 (0.89%)	145 (1.41%)	0.65	(0.42–1.02)	0.060	0.65	(0.42–1.01)	0.058

Follow-up years (mean ± SD) (the period between the HIV/AIDS diagnosis date and the neurological disease diagnosis date): 2.62 ± 2.31 years. N, number; INSTI, integrase strand transfer inhibitor; DDD, defined daily dose; ADH, adherence of ART drugs; OR, odds ratio; CI, confidence interval; Ref, reference. Model was applied using conditional logistic regression analysis adjusted by Charlson comorbidity number. Comorbidities were identified within the HIV/AIDS diagnosis date. Cumulative defined daily dose (DDD) and adherence (ADH) were calculated during the period between the HIV/AIDS diagnosis date and the neurological disease diagnosis date. The date of the first diagnosis of neurological disease was designated as the index date. The three categories included past exposure (2 < YEAR), recent exposure (1 < YEAR ≤ 2), and current exposure (0 < YEAR ≤ 1) according to the previous similar studies (PubMed PMID number: 31374345). Patients who received ART drugs during the time window of over 2 years before the index date but were currently non-users were defined as patients with past exposure. Patients who received ART drugs during the time window of 1–2 years before the index date were defined as patients with recent exposure. Patients who received ART drugs during the time window of 0–1 year before the index date were defined as patients with current exposure. Patients who had continuously received ART since their first diagnosis of HIV/AIDS were also defined as those with current exposure. The defined daily doses (DDDs) were those recommended by the Collaborating Center for Drug Statistics Methodology of the World Health Organization (WHO) (https://www.whocc.no/atc_ddd_index/). For the cumulative defined daily dose (DDD) of ART drugs, two categorized cumulative DDDs were defined according to (total amount of drug)/(amount of drug in a DDD) from the first date of ART treatment to the index date according to previous studies (PubMed PMID number: 31888519). For adherence (ADH) (electronic prescription claim data adherence) to ART drugs, two categorized ADHs were defined according to (total number of prescribed days)/(total number of observation days) from the first date of ART treatment to the index date according to previous studies (PubMed PMID number: 9681089). Significant *p*-values (*p* < 0.05) are highlighted in bold italic font.

**TABLE 7:** Odds ratios for neurological diseases when exposed to combinations of over two ART drugs per tablet.

Characteristics	Patients with neurological diseases	Patients with non-neurological diseases	Crude			Adjusted		
	(*N* = 2,571)	(*N* = 10,284)						
	*N* (%)	*N* (%)	OR	95% CI	*p*-value	OR	95% CI	*p*-value
Combination antiretroviral usage								
Non-exposure	804 (31.27%)	3,299 (32.08%)	1	Ref	Ref	1	Ref	Ref
Exposure	1767 (68.73%)	6,985 (67.92%)	1.05	(0.94–1.16)	0.392	1.04	(0.94–1.15)	0.438
Timing of exposure to combination antiretroviral drugs								
Non-exposure	804 (31.27%)	3,299 (32.08%)	1	Ref	Ref	1	Ref	Ref
Current exposure (0 < YEAR ≤ 1)	1,541 (59.94%)	5,990 (58.25%)	1.06	(0.96–1.18)	0.255	1.06	(0.95–1.17)	0.296
Recent exposure (1 < YEAR ≤ 2)	148 (5.76%)	754 (7.33%)	0.80	(0.66–0.98)	***0.033***	0.80	(0.66–0.98)	***0.033***
Past exposure (2 < YEAR)	78 (3.03%)	241 (2.34%)	1.34	(1.02–1.77)	***0.036***	1.34	(1.02–1.77)	***0.036***
Cumulative defined daily dose (DDD) of combination antiretroviral drugs								
Non-exposure	804 (31.27%)	3,299 (32.08%)	1	Ref	Ref	1	Ref	Ref
Cumulative DDDs < 823	1,068 (41.54%)	3,308 (32.17%)	1.36	(1.22–1.52)	***<0.001***	1.36	(1.22–1.52)	***<0.001***
Cumulative DDDs ≥ 823	699 (27.19%)	3,677 (35.75%)	0.63	(0.55–0.72)	***<0.001***	0.63	(0.55–0.72)	***<0.001***
Adherence (ADH) of combination antiretroviral drugs								
Non-exposure	804 (31.27%)	3,299 (32.08%)	1	Ref	Ref	1	Ref	Ref
Low (0 < ADH ≤ 0.8)	1,139 (44.3%)	3,705 (36.03%)	1.29	(1.15–1.44)	***<0.001***	1.29	(1.15–1.44)	***<0.001***
High (0.8 < ADH)	628 (24.43%)	3,280 (31.89%)	0.78	(0.68–0.88)	***<0.001***	0.77	(0.68–0.87)	***<0.001***

Follow-up years (mean ± SD) (the period between the HIV/AIDS diagnosis date and the neurological disease diagnosis date): 2.62 ± 2.31 years. N, number; DDD, defined daily dose; ADH, adherence of ART drugs; OR, odds ratio; CI, confidence interval; Ref, reference. Model was applied using conditional logistic regression analysis adjusted by Charlson comorbidity number. Comorbidities were identified within the HIV/AIDS diagnosis date. Cumulative defined daily dose (DDD) and adherence (ADH) were calculated during the period between the HIV/AIDS diagnosis date and the neurological disease diagnosis date. The date of the first diagnosis of neurological disease was designated as the index date. The three categories included past exposure (2 < YEAR), recent exposure (1 < YEAR ≤ 2), and current exposure (0 < YEAR ≤ 1) according to the previous similar studies (PubMed PMID number: 31374345). Patients who received ART drugs during the time window of over 2 years before the index date but were currently non-users were defined as patients with past exposure. Patients who received ART drugs during the time window of 1–2 years before the index date were defined as patients with recent exposure. Patients who received ART drugs during the time window of 0–1 year before the index date were defined as patients with current exposure. Patients who had continuously received ART since their first diagnosis of HIV/AIDS were also defined as those with current exposure. The defined daily doses (DDDs) were those recommended by the Collaborating Center for Drug Statistics Methodology of the World Health Organization (WHO) (https://www.whocc.no/atc_ddd_index/). For the cumulative defined daily dose (DDD) of ART drugs, two categorized cumulative DDDs were defined according to (total amount of drug)/(amount of drug in a DDD) from the first date of ART treatment to the index date according to previous studies (PubMed PMID number: 31888519). For adherence (ADH) (Electronic prescription claims data adherence) to ART drugs, two categorized ADHs were defined according to (total number of prescribed days)/(total number of observation days) from the first date of ART treatment to the index date according to previous studies (PubMed PMID number: 9681089). Significant *p*-values (*p* < 0.05) are highlighted in bold italic font.

## 3 Results

### 3.1 Demographic characteristics of study subjects

Between 2010 and 2017, 20,355 patients had HIV/AIDS (ICD-9-CM code: 042–044) (Figure 1). After excluding patients with no age or sex information, neurological diseases, malignancy, or hemiplegia before HIV/AIDS diagnosis, 17,335 patients were included in this study. A total of 2,594 patients with neurological diseases and 14,741 controls without neurological diseases were also included (Figure 1) (Table 1). Age, sex, comorbidities, ART usage, and follow-up period were significantly different between the two groups (total subjects; Table 1) (*p* < 0.05). Patients with neurological diseases were older, had a shorter follow-up period, and had an increased incidence of comorbidities (*p* < 0.05). However, the cases and controls showed no background differences after matching for age, sex, and first diagnosis date of HIV/AIDS, except for comorbidities between the two groups (matched subjects; Table 1). The matched patients with neurological diseases had more comorbidities than non-neurological disease controls.

### 3.2 Antiretroviral therapy (ART) and risk of neurological diseases

This study defined four types of neurological diseases (CNS infections, cognitive disorders, vasculopathy, and/or peripheral neuropathy) as outcomes (Tsai et al., 2017b). A conditional logistic regression model was used to investigate the association between antiretroviral therapy (ART) and the risk of neurological diseases in patients with HIV/AIDS (Table 2). Significant differences in the timing of exposure to ART, adherence to ART, cumulative central nervous system penetration effectiveness (CPE) score, and cumulative defined daily dose (DDD) (*p* < 0.05) were found.

For timing of exposure to ART, patients with past exposure to it presented an increased risk of neurological diseases, with an adjusted odds ratio (OR) of 1.68 (95% CI: 1.22–2.32) than those who did not use ART (*p* = 0.002) (Figure 2) (Table 2). No significant association was observed in patients with current or recent exposures (*p* > 0.05).

For cumulative DDD of ART, patients receiving higher cumulative DDDs (≥ 2,500) had a decreased risk of neurological diseases (adjusted OR, 0.53; 95% CI: 0.45–0.62) when compared with non-ART users (*p* < 0.001) (Table 2). However, patients receiving lower cumulative DDDs (< 2,500) showed a greater risk of neurological diseases (adjusted OR, 1.28; 95% CI: 1.15–1.42) than those not receiving ART (*p* < 0.001).

For adherence to ART, patients with higher adherence to the medication (> 0.8) had a lower risk of neurological diseases (adjusted OR, 0.78; 95% CI: 0.70–0.88) when compared with non-ART users (*p* < 0.001) (Table 2). However, patients with lower ART adherence (0 < ART adherence ≤ 0.8) had a higher risk of neurological diseases (adjusted OR, 1.46; 95% CI: 1.30–1.64) than those not receiving it (*p* < 0.001).

For cumulative CPE score, patients with a higher cumulative CPE score (> 18) had a higher risk of neurological diseases (adjusted OR, 1.33; 95% CI: 1.10–1.61) than those not receiving ART (*p* = 0.003) (Table 2). Patients with a cumulative CPE score of 14 < cumulative CPE score ≤ 18 had a greater risk of neurological diseases (adjusted OR, 1.34; 95% CI: 1.14–1.57) than those not receiving ART (*p* < 0.001). No significant associations were observed in patients with cumulative CPE scores less than 14 (*p* > 0.05). These results suggest that patients were at a greater risk of neurological diseases when they were only exposed to ART or received it with a greater cumulative CPE score (> 14) during the study period.

### 3.3 Classes of antiretroviral therapy (ART) drugs and risk of neurological diseases

ART drugs are classified into several categories based on their molecular mechanisms and pharmacological characteristics (Arts and Hazuda, 2012; Nastri et al., 2023). In our study, there were five main classes of ART drugs, i.e., NRTIs, PIs, NNRTIs, INSTIs, and combinations of over two ART drugs per tablet (Tables 3–7). To investigate which classes of ART drugs are associated with the risk of neurological diseases in our patients with HIV/AIDS in Taiwan, we also performed a conditional logistic regression according to various classes of ART drugs (Tables 3–7).

For NRTI drugs, patients had a higher risk of neurological diseases when they had a history of current exposure (odds ratio [OR]:1.26, 95% confidence interval [CI]:1.09–1.46) and low cumulative DDDs (< 564) (OR: 1.46, 95% CI: 1.27–1.68) (Table 3).

For PI drugs, patients had a higher risk of neurological diseases when they had a history of current exposure (odds ratio [OR]:1.20, 95% confidence interval [CI]:1.03–1.39), low cumulative DDDs (< 411) (OR: 1.41, 95% CI: 1.19–1.66), and low adherence (0 < adherence (ADH) ≤ 0.8) (OR: 1.29, 95% CI: 1.12–1.49) (Table 4).

For NNRTI drugs, patients had a higher risk of neurological diseases when they had a history of current exposure (odds ratio [OR]:1.13, 95% confidence interval [CI]:1.02–1.26), low cumulative DDDs (< 226) (OR: 1.37, 95% CI: 1.23–1.52), and low adherence (0 < adherence (ADH) ≤ 0.8) (OR: 1.18, 95% CI: 1.06–1.30) (Table 5).

For INSTI drugs, patients had a higher risk of neurological diseases when they had an exposure (odds ratio [OR]:1.25, 95% confidence interval [CI]:1.06–1.46), history of current exposure (OR: 1.38, 95% CI: 1.14–1.66), low cumulative DDDs (< 305) (OR: 1.64, 95% CI: 1.34–2.01), and low adherence [0 < adherence (ADH) ≤ 0.8] (OR: 1.38, 95% CI: 1.17–1.63) (Table 6).

For combinations of over two ART drugs per tablet, patients had a higher risk of neurological diseases when they had a history of past exposure (odds ratio [OR]:1.34, 95% confidence interval [CI]:1.02–1.77), low cumulative DDDs (< 823) (OR: 1.36, 95% CI: 1.22–1.52), and low adherence (0 < adherence (ADH) ≤ 0.8) (OR: 1.29, 95% CI: 1.15–1.44) (Table 7).

### 3.4 Antiretroviral therapy (ART) and the risk of CNS infections

We explored the frequency distribution of neurological disease subtypes in patients with neurological diseases (Supplementary Table S2). Among patients with neurological diseases, more than 64% of patients had CNS infections (1,681 of the 2,594 patients with neurological diseases) (Supplementary Table S2). Among patients with HIV/AIDS, more than 9% of patients had CNS infections (1,684 of a total of 17,335 subjects) (Supplementary Table S2).

A conditional logistic regression model was used to investigate the association between ART and the risk of CNS infections in these patients (Supplementary Table S3). Significant differences in the timing of exposure to ART, adherence to ART, cumulative CPE score, and cumulative DDD (*p* < 0.05) were found.

For the timing of exposure to ART, patients with past exposure to ART presented an increased risk of CNS infections, with an adjusted odds ratio (OR) of 2.26 (95% CI: 1.52–3.34) compared to those who did not use ART (*p* < 0.001) (Supplementary Table S3). No significant association was observed in patients with current or recent exposures (*p* > 0.05).

For cumulative DDD of ART, patients receiving high cumulative DDDs (≥ 2,500) had a decreased risk of CNS infections (adjusted OR, 0.44; 95% CI: 0.36–0.55) compared to non-ART users (*p* < 0.001) (Supplementary Table S3). However, patients receiving low cumulative DDDs (< 2,500) showed a greater risk of CNS infections (adjusted OR, 1.31; 95% CI: 1.15–1.48) than those not receiving ART (*p* < 0.001).

For adherence to the medication, patients with high ART adherence (> 0.8) had a lower risk of CNS infections (adjusted OR, 0.76; 95% CI: 0.66–0.88) than non-ART users (*p* < 0.001) (Supplementary Table S3). However, patients with low ART adherence (0 < ART adherence ≤ 0.8) had a higher risk of CNS infections (adjusted OR, 1.53; 95% CI: 1.33–1.76) than those not receiving ART (*p* < 0.001).

Regarding CPE, patients with cumulative CPE score > 18 had a higher risk of neurological diseases (adjusted OR, 1.39; 95% CI: 1.10–1.76) than those not receiving ART (*p* = 0.005) (Supplementary Table S3). These results suggest that patients were at a greater risk of CNS infections when they were only exposed to ART or received ART with a greater cumulative CPE score (> 18) during the study period.

### 3.5 Cumulative CPE score and risk of neurological diseases when stratified by cumulative DDD of ART

Our results showed that patients with increased cumulative CPE scores tended to have a greater risk of neurological diseases. Subgroup analyses were then stratified by cumulative DDD of ART [Figure 3A: patients with low cumulative DDDs (< 2,500); Figure 3B: patients with high cumulative DDDs (≥ 2,500)] and revealed the following.

Among patients with low cumulative DDDs (< 2,500), those with the highest cumulative CPE score (> 18) had a higher risk of neurological diseases (OR, 1.86; 95% CI: 1.48–2.34) than those not receiving ART (*p* = 1.00E-07) (Figure 3A). Patients with a greater cumulative CPE score (14 < cumulative CPE score ≤ 18) had a greater risk of neurological diseases (OR, 1.56; 95% CI: 1.31–1.85) than those not receiving ART (*p* = 5.00E-07). Patients with a lower cumulative CPE score (10 < cumulative CPE score ≤ 14) had a higher risk of neurological diseases (OR, 1.23; 95% CI: 1.05–1.44) than those not receiving ART (*p* = 0.009). Patients with the lowest cumulative CPE score (0 < cumulative CPE score ≤ 10) had a higher risk of neurological diseases (OR, 1.14; 95% CI: 1.02–1.28) than those not receiving ART (*p* = 0.023).

Patients with high cumulative DDDs (≥ 2,500) had the lowest cumulative CPE score (0 < cumulative CPE score ≤ 10) and had the lowest risk of neurological diseases (OR, 0.51; 95% CI: 0.40–0.65), compared to those not receiving ART (*p* = 1.00E-08) (Figure 3B). Patients with a decreased cumulative CPE score (10 < cumulative CPE score ≤ 14) had a lower risk of neurological diseases (OR, 0.68; 95% CI: 0.56–0.84) than those not receiving ART (*p* = 3.00E-04). No significant associations were observed in patients with cumulative CPE scores of > 14 (*p* > 0.05). These results suggest that patients were at a greater risk of neurological diseases when they had lower cumulative DDDs (< 2,500) and greater cumulative CPE scores during the study period.

### 3.6 Cumulative CPE score and risk of neurological diseases when stratified by adherence to ART

Similarly, subgroup analyses were also performed stratifying the patients by their adherence to ART [Figure 4A: patients with low adherence (0 < ART adherence ≤ 0.8); Figure 4B: patients with high ART adherence (> 0.8)]. For patients with low adherence (0 < ART adherence ≤ 0.8), those with the highest cumulative CPE score (> 18) had a higher risk of neurological diseases (OR, 1.85; 95% CI: 1.49–2.31) than those not receiving ART (*p* = 2.00E-08) (Figure 4A). Patients with a greater cumulative CPE score (14 < cumulative CPE score ≤ 18) had a greater risk of neurological diseases (OR, 1.78; 95% CI: 1.46–2.16) than those not receiving ART (*p* = 6.00E-09). Patients with a decreased cumulative CPE score (10 < cumulative CPE score ≤ 14) had a greater risk of neurological diseases (OR, 1.31; 95% CI: 1.10–1.56) than those not receiving ART (*p* = 0.003). Patients with the lowest cumulative CPE score (0 < cumulative CPE score ≤ 10) had a greater risk of neurological diseases (OR, 1.35; 95% CI: 1.19–1.53) than those not receiving ART (*p* = 5.00E-06).

Patients with high ART adherence (> 0.8) had the lowest cumulative CPE score (0 < cumulative CPE score ≤ 10) and a lower risk of neurological diseases (OR, 0.76; 95% CI: 0.66–0.87) than those who did not receive ART (*p* = 7.00E-05) (Figure 4B). Patients with a lower cumulative CPE score (10 < cumulative CPE score ≤ 14) had a lower risk of neurological diseases (OR, 0.78; 95% CI: 0.66–0.93) than those who did not (*p* = 0.005). No significant associations were observed in patients with cumulative CPE scores of > 14 (*p* > 0.05). These results suggest that patients were at an elevated risk of neurological diseases when they had lower ART adherence (0 < ART adherence ≤ 0.8) and higher cumulative CPE scores during the study period.

## 4 Discussion

In this nested case-control study, we explored the effects of ART on the risk of neurological diseases in patients with HIV-1/AIDS in Taiwan. We found that patients had a higher risk of neurological diseases when they had a history of past exposure (> 2 years ago), low cumulative DDDs (< 2,500), low adherence (0 < adherence (ADH) ≤ 0.8), and high cumulative CPE scores (> 14) during the follow-up period (2.62 years). When stratified by classes of ART drugs, patients with low cumulative DDDs or low adherence had a high risk of neurological diseases, including those associated with NRTIs, PIs, NNRTIs, INSTIs, and combinations of more than two ART drugs per tablet. Furthermore, subgroup analyses showed that patients with low cumulative DDDs or low adherence had a higher risk of neurological diseases when they had higher cumulative CPE scores, in a dose-dependent manner. Interestingly, patients with high cumulative DDDs or medication adherence were protected against neurological diseases only when they received lower cumulative CPE scores (≤ 14) during the follow-up period. Therefore, our nested case-control study suggests that patients may be at risk of neurological diseases when they interrupt ART, have low cumulative DDDs, low adherence, or usage with high cumulative CPE scores. Continuous usage and lower cumulative CPE scores of ART drugs may benefit neurocognitive health in patients with HIV/AIDS.

Studies have explored the association between various categories of ART drugs, such as NRTIs, NNRTIs, PIs, and INSTIs, and neurotoxicity (Blanche et al., 1999; Cepeda and Wilks, 2000; Streck et al., 2008; Streck et al., 2011; Abers et al., 2014; Underwood et al., 2015; Hoffmann and Llibre, 2019; Zash et al., 2019; Foster et al., 2022); however, their relationship remains to be elucidated. In this study, we investigated which classes of ART drugs are associated with the risk of neurological diseases in patients with HIV/AIDS in Taiwan and observed that low cumulative DDDs or poor adherence to ART drugs increase the risk of neurological diseases across various classes of drugs, including NRTIs, PIs, NNRTIs, INSTIs, and multi-drug tablets. HIV is usually initially transmitted through the exchange of infected body fluids from infected individuals (Kordy et al., 2019). HIV continues to infect and replicates in T lymphocytes and monocyte-derived macrophages in the bloodstream and lymphoid tissues, leading to viremia (Naif, 2013). HIV can transit via endothelial cells directly or use the Trojan horse strategy via infected T cells or monocytes/macrophages (Hazleton et al., 2010; Rojas-Celis et al., 2019). Furthermore, large amounts of HIV, viral proteins, and inflammatory cytokines circulate in the bloodstream and damage the integrity and function of the blood–brain barrier (BBB) (Atluri et al., 2015; McRae, 2016). BBB dysfunction can lead to the entry of HIV-1 infected cells into the brain, increase the permeability of the barrier to small water-soluble compounds, and affect the ability of therapeutic drugs to penetrate the brain (Mahajan et al., 2008). Our results suggest that the harm caused by the virus itself in the BBB and CNS system could be more severe and long-lasting than the damage caused by ART-induced neurotoxicity. Taking ART drugs earlier and on a continuous basis may be beneficial for preserving cognitive function.

Studies have shown contradictory results between CPE scores and the risk of neurocognitive impairment in HIV/AIDS (Marra et al., 2009; Smurzynski et al., 2011; Liao and Tsai, 2013; Caniglia et al., 2014; Vassallo et al., 2014; Carvalhal et al., 2016). Carvalhal et al. (2016) reported that higher CPE scores correlated significantly with a lower prevalence of neurocognitive impairment. Vassallo et al. (2014) reported that a combination antiretroviral therapy with better CSF penetration could protect against cognitive deterioration. Smurzynski et al. (2011) also reported that the use of antiretroviral drugs with better estimated CNS penetration may be associated with better neurocognitive functioning. However, our results showed that higher cumulative CPE scores were significantly correlated with a higher risk of neurological diseases. Patients with high cumulative DDDs or high adherence to medication were protected against neurological diseases only when they received lower cumulative CPE scores (≤ 14). Our results are in agreement with those of previous studies (Marra et al., 2009; Caniglia et al., 2014). Marra et al. (2009) reported that antiretrovirals with good CNS penetration are associated with poorer neurocognitive performance. Caniglia et al. (2014) also reported that a high CPE score increased the risk of HIV dementia.

We observed that higher cumulative ART doses and adherence were protective against neurological disease. Only patients with a history of exposure, low cumulative DDDs, and low adherence had an increased risk of neurological diseases. Our results may agree with those of similar studies suggesting that neurocognitive impairment is associated with HIV-1 virus-induced neurotoxicity and immune suppression in the central nervous system (CNS) in patients with low adherence to combination ART (Brew et al., 1997; Kamal et al., 2017; Ruhanya et al., 2022; Wallace, 2022).

Each antiretroviral drug was assigned a CPE score (Caniglia et al., 2014; Nwogu et al., 2016; Santos et al., 2019; Lanman et al., 2021). The antiretroviral drugs with the highest CPE score (level 4) were dolutegravir, indinavir, nevirapine, and zidovudine. Three of these are associated with neurocognitive functions (Huang et al., 2017; Wu et al., 2017; Montenegro-Burke et al., 2019; Allen Reeves et al., 2021). In a rodent model, Montenegro-Burke et al. (2019) reported that dolutegravir induces metabolic oxidative stress within the cerebellum and frontal cortex. In a meta-analysis of patients with HIV/AIDS, Allen Reeves et al. (2021) reported that the combination of dolutegravir/rilpivirine increases the risk of depressive symptoms. Huang et al. (2017) reported that indinavir induces peripheral neuropathy in a rodent model. Wu et al. (2017) reported that zidovudine induces proinflammatory cytokines via Wnt5a in the CNS of a mouse model. Therefore, our nested case-control results indicate that the continuous use of ART drugs with lower cumulative CPE scores may benefit neurocognitive health in patients with HIV/AIDS.

The limitations of this study include the lack of human genetic factors, environmental factors (nutrition, education, job stress, and exercise), and clinical characteristics (blood physiological and biochemical measures in this database, including CD4 counts, CD4 nadir, CD4/CD8, body mass index, fatty tissue, and cigarette smoking).

Patients with a history of past exposure, low cumulative DDDs, low adherence, and high cumulative CPE scores were at a higher risk of neurological diseases. Patients with high cumulative DDDs or adherence were protected against neurological diseases only when they had a lower cumulative CPE score. This study provides evidence that continuous usage and lower cumulative CPE scores of ART drugs may benefit neurocognitive health in patients with HIV/AIDS.

## Data Availability

The data analyzed in this study is subject to the following licenses/restrictions: Only citizens of the Republic of China who fulfill the requirements of conducting research projects are eligible to apply for the National Health Insurance Research Database (NHIRD). The use of NHIRD is limited to research purposes only. Applicants must follow the Computer-Processed Personal Data Protection Law (http://www.winklerpartners.com/?p=987) and related regulations of National Health Insurance Administration and NHRI (National Health Research Institutes), and an agreement must be signed by the applicant and his/her supervisor upon application submission. All applications are reviewed for approval of data release. Requests to access these datasets should be directed to Y-JL, yjlin.kath@gmail.com.

## Abbreviations

ADH, adherence; ART, antiretroviral therapy; BBB, blood–brain barrier; CCI, Charlson comorbidity index; CI, confidence interval; CNS, central nervous system; CPE, CNS penetration effectiveness; CSF, cerebrospinal fluid; DDD, defined daily dose; HAART, highly active antiretroviral therapy; ICD-9-CM, International Classification of Diseases, Ninth Revision, Clinical Modification; NRTI, nucleoside reverse-transcriptase inhibitors; NHIRD, National Health Insurance Research Database; OR, odds ratio; PI, protease inhibitor.
