# Supplementary material for: Association of combination antiretroviral therapy with risk of neurological diseases in patients with HIV/AIDS in Taiwan: a nested case-control study
Source: Front Pharmacol. 2023 Jun 8;14:1110605. doi: 10.3389/fphar.2023.1110605 (PMC10285306; doi:10.3389/fphar.2023.1110605)
Supplement: Supplementary file 1 [file Table1.DOCX]

**Supplementary Information**

Supplementary Text

**Tables S1-S5**

**TABLE S1** | Number and percent distribution of inpatient and outpatient visits within one year after the diagnosis of HIV/AIDS.

**TABLE S2 |** Neurological disease distribution of patients with HIV/AIDS in Taiwan.

**TABLE S3 |** Odds ratios for CNS infections when exposure to ART drugs.

**TABLE S4 |** Odds ratios for peripheral neuropathy when exposure to NRTI drugs.

**TABLE S5 |** Frequency distribution of substance use disorders, HBV, HCV, and syphilis infections in patients with HIV/AIDS in Taiwan.

| **TABLE S1 \| Number and percent distribution of inpatient and outpatient visits within one year after the diagnosis of HIV/AIDS** | | | |
| --- | --- | --- | --- |
| **No.** | **Type of clinic** | **Number of visits** | **Percentage (number of visits)** |
| **1** | Infectious Disease | 714 | 50.53 |
| **2** | Internal Medicine | 235 | 16.63 |
| **3** | Emergency Medicine | 96 | 6.79 |
| **4** | Psychiatry | 67 | 4.74 |
| **5** | Thoracic Medicine | 36 | 2.55 |
| **6** | Neurology | 34 | 2.41 |
| **7** | Dermatology | 26 | 1.84 |
| **8** | Surgery | 21 | 1.49 |
| **9** | Colorectal Surgery | 21 | 1.49 |
| **10** | Gastroenterology | 20 | 1.42 |
| **11** | Ophthalmology | 16 | 1.13 |
| **12** | Orthopedic | 13 | 0.92 |
| **13** | Otorhinolaryngology | 12 | 0.85 |
| **14** | Rheumatology and Immunology | 12 | 0.85 |
| **15** | Neurosurgery | 10 | 0.71 |
| **16** | Family Medicine | 9 | 0.64 |
| **17** | Urology | 8 | 0.57 |
| **18** | Rehabilitation | 8 | 0.57 |
| **19** | Nephrology | 7 | 0.5 |
| **20** | Gastrointestinal Surgery | 7 | 0.5 |
| **21** | Tuberculosis | 6 | 0.42 |
| **22** | Cardiology and Vascular Medicine | 5 | 0.35 |
| **23** | Thoracic Surgery | 5 | 0.35 |
| **24** | Obstetrics and Gynecology | 3 | 0.21 |
| **25** | Thoracic and Critical Care Medicine | 3 | 0.21 |
| **26** | Home Care | 3 | 0.21 |
| No., number. | | | |
| Total visits = 1413 within one year after the diagnosis of HIV/AIDS. | | | |

| **TABLE S2 \| Neurological disease distribution of patients with HIV/AIDS in Taiwan** | | | | | | |
| --- | --- | --- | --- | --- | --- | --- |
| **Characteristics** | **Total subjects** | | ***p*-value** | **Matched subjects** | | ***p*-value** |
|  | **Patients with neurological diseases** | **Patients with non-neurological diseases** |  | **Patients with neurological diseases** | **Patients with non-neurological diseases** |  |
|  | **(N = 2,594)** | **(N = 14,741)** |  | **(N = 2,571)** | **(N = 10,284)** |  |
|  | **N (%)** | **N (%)** |  | **N (%)** | **N (%)** |  |
| CNS infections |  |  | ***<0.001*** |  |  | ***<0.001*** |
| No | 913 (35.20%) | 14741 (100%) |  | 932 (36.25%) | 10284 (100%) |  |
| Yes | 1681 (64.80%) | 0 (0.00%) |  | 1639 (63.75%) | 0 (0.00%) |  |
| Cognitive disorders |  |  | ***<0.001*** |  |  | ***<0.001*** |
| No | 2226 (85.81%) | 14741 (100%) |  | 2215 (86.15%) | 10284 (100%) |  |
| Yes | 368 (14.19%) | 0 (0.00%) |  | 356 (13.85%) | 0 (0.00%) |  |
| Vasculopathy |  |  | ***<0.001*** |  |  | ***<0.001*** |
| No | 2433 (93.79%) | 14741 (100%) |  | 2417 (94.01%) | 10284 (100%) |  |
| Yes | 161 (6.21%) | 0 (0.00%) |  | 154 (5.99%) | 0 (0.00%) |  |
| Peripheral neuropathy |  |  | ***<0.001*** |  |  | ***<0.001*** |
| No | 2179 (84.00%) | 14741 (100%) |  | 2170 (84.40%) | 10284 (100%) |  |
| Yes | 415 (16.00%) | 0 (0.00%) |  | 401 (15.60%) | 0 (0.00%) |  |
| N, number; CNS, central nervous system. | | | | | | |
| Neurological diseases were identified during the study period. | | | | | | |
| The ICD-9-CM codes: (1) CNS infections (ICD-9-CM codes: 013, 047, 053, 094, 200, 320, 321, 322, 323, 003.21, 054.3, 054.4, 098.82, 112.83, 114.2, 115.91, 130.0, and 321.0); (2) cognitive disorders (ICD-9-CM codes: 290, 293, 294, 332, 345, 348.1, 348.3, and 780.3); (3) vasculopathy (ICD-9-CM codes: 325, 430, 431, 432, 433, 434, 435, 436, and 437); and (4) peripheral neuropathy (ICD-9-CM codes: 350, 351, 353, 354, 355, 356, 357, and 358). | | | | | | |

| **TABLE S3 \| Odds ratios for CNS infections when exposure to ART drugs** | | | | | | | | | |
| --- | --- | --- | --- | --- | --- | --- | --- | --- | --- |
| **Characteristics** | **Patients with CNS infections** | **Patients with non-CNS infections** | **Crude** | | |  | **Adjusted** | | |
|  | **(N =1738)** | **(N = 6952)** |  |  |  |  |  |  |  |
|  | **N (%)** | **N (%)** | **OR** | **95% CI** | ***p*-value** |  | **OR** | **95% CI** | ***p*-value** |
| **ART usage** |  |  |  |  |  |  |  |  |  |
| Non-ART use | 495 (28.48%) | 2100 (30.21%) | 1 | Ref | Ref |  | 1 | Ref | Ref |
| ART use | 1243 (71.52%) | 4852 (69.79%) | 1.10 | (0.97-1.25) | 0.125 |  | 1.10 | (0.97-1.25) | 0.133 |
| **Timing of exposure to ART drugs** |  |  |  |  |  |  |  |  |  |
| Non-ART use | 495 (28.48%) | 2100 (30.21%) | 1 | Ref | Ref |  | 1 | Ref | Ref |
| Current exposure (0 < YEAR ≤1) | 1116 (64.21%) | 4326 (62.23%) | 1.11 | (0.98-1.26) | 0.108 |  | 1.11 | (0.97-1.26) | 0.117 |
| Recent exposure (1 < YEAR ≤2) | 84 (4.83%) | 442 (6.36%) | 0.81 | (0.62-1.06) | 0.133 |  | 0.81 | (0.62-1.06) | 0.127 |
| Past exposure (2 < YEAR) | 43 (2.47%) | 84 (1.21%) | 2.25 | (1.52-3.33) | ***<0.001*** |  | 2.26 | (1.52-3.34) | ***<0.001*** |
| **Cumulative defined daily dose (DDD) of ART drugs** |  |  |  |  |  |  |  |  |  |
| Non-ART use | 495 (28.48%) | 2100 (30.21%) | 1 | Ref | Ref |  | 1 | Ref | Ref |
| Cumulative DDDs < 2500 | 1029 (59.21%) | 3437 (49.44%) | 1.31 | (1.15-1.49) | ***<0.001*** |  | 1.31 | (1.15-1.48) | ***<0.001*** |
| Cumulative DDDs ≥ 2500 | 214 (12.31%) | 1415 (20.35%) | 0.45 | (0.36-0.55) | ***<0.001*** |  | 0.44 | (0.36-0.55) | ***<0.001*** |
| **Adherence (ADH) of ART drugs** |  |  |  |  |  |  |  |  |  |
| Non-ART use | 495 (28.48%) | 2100 (30.21%) | 1 | Ref | Ref |  | 1 | Ref | Ref |
| Low (0 < ADH ≤ 0.8) | 738 (42.46%) | 2087 (30.02%) | 1.53 | (1.33-1.76) | ***<0.001*** |  | 1.53 | (1.33-1.76) | ***<0.001*** |
| High (0.8 < ADH) | 505 (29.06%) | 2765 (39.77%) | 0.76 | (0.66-0.89) | ***<0.001*** |  | 0.76 | (0.66-0.88) | ***<0.001*** |
| **Cumulative CPE score of ART drugs** |  |  |  |  |  |  |  |  |  |
| Non-ART use | 495 (28.48%) | 2100 (30.21%) | 1 | Ref | Ref |  | 1 | Ref | Ref |
| 0 ≦ Cumulative CPE score ≦ 10 | 638 (36.71%) | 2563 (36.87%) | 1.07 | (0.93-1.23) | 0.324 |  | 1.07 | (0.93-1.23) | 0.335 |
| 10 < Cumulative CPE score ≦14 | 267 (15.36%) | 1109 (15.95%) | 1.06 | (0.89-1.26) | 0.539 |  | 1.05 | (0.88-1.26) | 0.553 |
| 14 < Cumulative CPE score ≦18 | 197 (11.33%) | 726 (10.44%) | 1.20 | (0.99-1.47) | 0.069 |  | 1.20 | (0.98-1.46) | 0.074 |
| 18 < Cumulative CPE score | 141 (8.11%) | 454 (6.53%) | 1.40 | (1.11-1.77) | ***0.005*** |  | 1.39 | (1.10-1.76) | ***0.005*** |
| Follow-up years (Mean±SD) (the period between the diagnosed date of HIV/AIDS and the diagnosed date of neurological diseases): 2.62 ± 2.31 years. | | | | | | | | | |
| N, number; ART, antiretroviral therapy; DDD, defined daily dose; ADH, adherence of ART drugs; CPE score, central nervous system penetration effectiveness (CPE) score; OR, odds ratio; CI, confidence interval; Ref, reference. | | | | | | | | | |
| Model was applied using conditional logistic regression analysis adjusted by Charlson comorbidity number. Comorbidities were identified within the diagnosed date of HIV/AIDS. | | | | | | | | | |
| Cumulative defined daily dose (DDD), adherence (ADH), and central nervous system penetration effectiveness (CPE) score were calculated during the period between the diagnosted date of HIV/AIDS and the diagnosed date of neurological diseases. | | | | | | | | | |
| The date of the first diagnosis of neurological disease was designated as the index date. The three categories included past exposure (2 < YEAR), recent exposure (1 < YEAR ≤ 2), and current exposure (0 < YEAR ≤ 1) according to the previous similar studies (PubMed PMID number: 31374345). Patients who received ART drugs during the time window of over 2 years before the index date, but current non-use were defined as patients with past exposure. Patients who received ART drugs during the time window of 1–2 years before the index date were defined as patients with recent exposure. Patients who received ART drugs during the time window of 0–1 year before the index date were defined as patients with current exposure. Patients who had continuously received ART since their first diagnosis of HIV/AIDS were also defined as those with current exposure. | | | | | | | | | |
| The defined daily doses (DDDs) were those recomended by the Collaborating Center for Drug Statistics Methodology of the World Health Organization (WHO) (https://www.whocc.no/atc_ddd_index/). For the cumulative defined daily dose (DDD) of ART drugs, two categorized cumulative DDDs were defined according to (total amount of drug)/(amount of drug in a DDD) from the first date of ART treatment to the index date according to previous studies (PubMed PMID number: 31888519). | | | | | | | | | |
| For adherence (ADH) (Electronic prescription claims data adherence) to ART drugs, two categorized ADHs were defined according to (total number of prescribed days) / (total number of observation days) from the first date of ART treatment to the index date according to previous studies (PubMed PMID number: 9681089). | | | | | | | | | |
| For the central nervous system penetration effectiveness (CPE) score, four categorized CPE scores were defined according to the sum of the ranks of each ART drug in the regimen administered to a patient from the start of drug usage to the first diagnosis date of neurological diseases (Table 2). The rank of each ART drug was determined according to previous studies (PubMed PMID number: 24907236, 31304188, 31823251, and 31385157). The CPE score for a particular regimen was determined by adding together the rankings of each ART drug included in the regimen. If the regimen was altered, the ranking of the newly added ART drug was added to the accumulated CPE score during the study period. | | | | | | | | | |
| Significant *p*-values (*p* < 0.05) are highlighted in bold italic font. | | | | | | | | | |

| **TABLE S4 \| Odds ratios for peripheral neuropathy when exposure to NRTI drugs** | | | | | | | | | |
| --- | --- | --- | --- | --- | --- | --- | --- | --- | --- |
| **Characteristics** | **Patients with peripheral neuropathy** | **Patients with non-peripheral neuropathy** | **Crude** | | |  | **Adjusted** | | |
|  | **(N =479)** | **(N = 1916)** |  |  |  |  |  |  |  |
|  | **N (%)** | **N (%)** | **OR** | **95% CI** | ***p*-value** |  | **OR** | **95% CI** | ***p*-value** |
| **NRTI usage** |  |  |  |  |  |  |  |  |  |
| Non-exposure | 360 ( 75.16%) | 1461 ( 76.25%) | 1 | Ref | Ref |  | 1 | Ref | Ref |
| Exposure | 119 ( 24.84%) | 455 ( 23.75%) | 1.06 | (0.84-1.35) | 0.608 |  | 1.05 | (0.83-1.33) | 0.680 |
| **Timing of exposure to NRTI drugs** |  |  |  |  |  |  |  |  |  |
| Non-exposure | 360 ( 75.16%) | 1461 ( 76.25%) | 1 | Ref | Ref |  | 1 | Ref | Ref |
| Current exposure (0 < YEAR ≤1) | 56 ( 11.69%) | 189 ( 9.86%) | 1.21 | (0.87-1.68) | 0.254 |  | 1.20 | (0.86-1.66) | 0.290 |
| Recent exposure (1 < YEAR ≤2) | 15 ( 3.13%) | 83 ( 4.33%) | 0.72 | (0.41-1.29) | 0.270 |  | 0.72 | (0.40-1.28) | 0.259 |
| Past exposure (2 < YEAR) | 48 ( 10.02%) | 183 ( 9.55%) | 1.06 | (0.74-1.51) | 0.765 |  | 1.04 | (0.73-1.49) | 0.817 |
| **Cumulative defined daily dose (DDD) of NRTI drugs** |  |  |  |  |  |  |  |  |  |
| Non-exposure | 360 ( 75.16%) | 1461 ( 76.25%) | 1 | Ref | Ref |  | 1 | Ref | Ref |
| Cumulative DDDs < 612 | 65 ( 13.57%) | 224 ( 11.69%) | 1.17 | (0.87-1.58) | 0.290 |  | 1.16 | (0.86-1.56) | 0.337 |
| Cumulative DDDs ≥ 612 | 54 ( 11.27%) | 231 ( 12.06%) | 0.95 | (0.68-1.31) | 0.741 |  | 0.94 | (0.67-1.30) | 0.702 |
| **Adherence (ADH) of NRTI drugs** |  |  |  |  |  |  |  |  |  |
| Non-exposure | 360 ( 75.16%) | 1461 ( 76.25%) | 1 | Ref | Ref |  | 1 | Ref | Ref |
| Low (0 < ADH ≤ 0.8) | 107 ( 22.34%) | 367 ( 19.15%) | 1.19 | (0.93-1.53) | 0.175 |  | 1.17 | (0.91-1.51) | 0.207 |
| High (0.8 < ADH) | 12 ( 2.51%) | 88 ( 4.59%) | 0.55 | (0.29-1.02) | 0.057 |  | 0.54 | (0.29-1.00) | 0.051 |
| Follow-up years (Mean±SD) (the period between the diagnosed date of HIV/AIDS and the diagnosed date of neurological diseases): 2.62 ± 2.31 years. | | | | | | | | | |
| N, number; NRTI, nucleoside reverse-transcriptase inhibitors; DDD, defined daily dose; ADH, adherence of ART drugs; OR, odds ratio; CI, confidence interval; Ref, reference. | | | | | | | | | |
| Model was applied using conditional logistic regression analysis adjusted by Charlson comorbidity number. Comorbidities were identified within the diagnosed date of HIV/AIDS. | | | | | | | | | |
| Cumulative defined daily dose (DDD) and adherence (ADH) were calculated during the period between the diagnosted date of HIV/AIDS and the diagnosed date of neurological diseases. | | | | | | | | | |
| The date of the first diagnosis of neurological disease was designated as the index date. The three categories included past exposure (2 < YEAR), recent exposure (1 < YEAR ≤ 2), and current exposure (0 < YEAR ≤ 1) according to the previous similar studies (PubMed PMID number: 31374345). Patients who received ART drugs during the time window of over 2 years before the index date, but current non-use were defined as patients with past exposure. Patients who received ART drugs during the time window of 1–2 years before the index date were defined as patients with recent exposure. Patients who received ART drugs during the time window of 0–1 year before the index date were defined as patients with current exposure. Patients who had continuously received ART since their first diagnosis of HIV/AIDS were also defined as those with current exposure. | | | | | | | | | |
| The defined daily doses (DDDs) were those recomended by the Collaborating Center for Drug Statistics Methodology of the World Health Organization (WHO) (https://www.whocc.no/atc_ddd_index/). For the cumulative defined daily dose (DDD) of ART drugs, two categorized cumulative DDDs were defined according to (total amount of drug)/(amount of drug in a DDD) from the first date of ART treatment to the index date according to previous studies (PubMed PMID number: 31888519). | | | | | | | | | |
| For adherence (ADH) (Electronic prescription claims data adherence) to ART drugs, two categorized ADHs were defined according to (total number of prescribed days) / (total number of observation days) from the first date of ART treatment to the index date according to previous studies (PubMed PMID number: 9681089). | | | | | | | | | |
| Significant *p*-values (*p* < 0.05) are highlighted in bold italic font. | | | | | | | | | |

| **TABLE S5 \| Frequency distribution of substance use disorders, HBV, HCV, and syphilis infections in patients with HIV/AIDS in Taiwan** | | | | | | |
| --- | --- | --- | --- | --- | --- | --- |
| **Characteristics** | **Total subjects** | | ***p*-value** | **Matched subjects** | | ***p*-value** |
|  | **Patients with neurological diseases** | **Patients with non-neurological diseases** |  | **Patients with neurological diseases** | **Patients with non-neurological diseases** |  |
|  | **(N = 2,594)** | **(N = 14,741)** |  | **(N = 2,571)** | **(N = 10,284)** |  |
|  | **N (%)** | **N (%)** |  | **N (%)** | **N (%)** |  |
| Substance use disorders (F10-F19) |  |  | <0.001 |  |  | 0.988 |
| No | 2369 ( 91.33%) | 13818 ( 93.74%) |  | 2347 ( 91.29%) | 9387 ( 91.28%) |  |
| Yes | 225 ( 8.67%) | 923 ( 6.26%) |  | 224 ( 8.71%) | 897 ( 8.72%) |  |
| HBV, HCV infection (B15-B19) |  |  | 0.812 |  |  | 0.894 |
| No | 2449 ( 94.41%) | 13934 ( 94.53%) |  | 2426 ( 94.36%) | 9697 ( 94.29%) |  |
| Yes | 145 ( 5.59%) | 807 ( 5.47%) |  | 145 ( 5.64%) | 587 ( 5.71%) |  |
| Syphilis, unspecified (**A50-**A53) |  |  | 0.027 |  |  | 0.935 |
| No | 2144 ( 82.65%) | 11912 ( 80.81%) |  | 2123 ( 82.57%) | 8485 ( 82.51%) |  |
| Yes | 450 ( 17.35%) | 2829 ( 19.19%) |  | 448 ( 17.43%) | 1799 ( 17.49%) |  |
| N, number; HBV, hepatitis B virus; HCV, hepatitis C virus. | | | | | | |
| Substance use disorder, HBV, HCV, and syphilis infections present within the date of HIV infection diagnosis. | | | | | | |
